# Lung adenocarcinoma promotion by air pollutants

**DOI:** 10.1038/s41586-023-05874-3

**Published:** 2023-04-05

**Authors:** William Hill, Emilia L. Lim, Clare E. Weeden, Claudia Lee, Marcellus Augustine, Kezhong Chen, Feng-Che Kuan, Fabio Marongiu, Edward J. Evans, David A. Moore, Felipe S. Rodrigues, Oriol Pich, Bjorn Bakker, Hongui Cha, Renelle Myers, Febe van Maldegem, Jesse Boumelha, Selvaraju Veeriah, Andrew Rowan, Cristina Naceur-Lombardelli, Takahiro Karasaki, Monica Sivakumar, Swapnanil De, Deborah R. Caswell, Ai Nagano, James R. M. Black, Carlos Martínez-Ruiz, Min Hyung Ryu, Ryan D. Huff, Shijia Li, Marie-Julie Favé, Alastair Magness, Alejandro Suárez-Bonnet, Simon L. Priestnall, Margreet Lüchtenborg, Katrina Lavelle, Joanna Pethick, Steven Hardy, Fiona E. McRonald, Meng-Hung Lin, Clara I. Troccoli, Moumita Ghosh, York E. Miller, Daniel T. Merrick, Robert L. Keith, Maise Al Bakir, Chris Bailey, Mark S. Hill, Lao H. Saal, Yilun Chen, Anthony M. George, Christopher Abbosh, Nnennaya Kanu, Se-Hoon Lee, Nicholas McGranahan, Christine D. Berg, Peter Sasieni, Richard Houlston, Clare Turnbull, Stephen Lam, Philip Awadalla, Eva Grönroos, Julian Downward, Tyler Jacks, Christopher Carlsten, Ilaria Malanchi, Allan Hackshaw, Kevin Litchfield, TRACERx Consortium, James DeGregori, Mariam Jamal-Hanjani, Charles Swanton

**Affiliations:** 1 Cancer Evolution and Genome Instability Laboratory, The Francis Crick Institute, London, UK; 2 Cancer Research UK Lung Cancer Centre of Excellence, University College London Cancer Institute, London, UK; 3 Division of Medicine, University College London, London, UK; 4 Tumour Immunogenomics and Immunosurveillance Laboratory, University College London Cancer Institute, London, UK; 5 Department of Thoracic Surgery and Thoracic Oncology Institute, Peking University People's Hospital, Beijing, China; 6 Department of Hematology and Oncology, Chang Gung Memorial Hospital, Chiayi Branch, Chiayi, Taiwan; 7 Graduate Institute of Clinical Medical Sciences, Chang-Gung University, Taoyuan, Taiwan; 8 Department of Biochemistry and Molecular Genetics, University of Colorado Anschutz Medical Campus, Aurora, CO, USA; 9 Department of Biomedical Sciences, University of Cagliari, Cagliari, Italy; 10 Department of Cellular Pathology, University College London Hospitals, London, UK; 11 Tumour-Host Interaction Laboratory, The Francis Crick Institute, London, UK; 12 Division of Hematology-Oncology, Department of Medicine, Samsung Medical Center, Sungkyunkwan University School of Medicine, Seoul, Korea; 13 BC Cancer Research Institute, University of British Columbia, Vancouver, British Columbia, Canada; 14 Oncogene Biology Laboratory, The Francis Crick Institute, London, UK; 15 Department of Molecular Cell Biology and Immunology, Amsterdam UMC, Amsterdam, The Netherlands; 16 Cancer Metastasis Laboratory, University College London Cancer Institute, London, UK; 17 Cancer Genome Evolution Research Group, Cancer Research UK Lung Cancer Centre of Excellence, University College London Cancer Institute, London, UK; 18 Department of Medicine, Division of Respiratory Medicine, Chan-Yeung Centre for Occupational and Environmental Respiratory Disease, Vancouver Coastal Health Research Institute, UBC, Vancouver, British Columbia, Canada; 19 Ontario Institute for Cancer Research, Toronto, Ontario, Canada; 20 Department of Pathobiology and Population Sciences, The Royal Veterinary College, Hatfield, UK; 21 Experimental Histopathology, The Francis Crick Institute, London, UK; 22 National Disease Registration Service (NDRS), NHS England, Leeds, UK; 23 Centre for Cancer, Society and Public Health, Comprehensive Cancer Centre, School of Cancer and Pharmaceutical Sciences, King's College London, London, UK; 24 Health Information and Epidemiology Laboratory, Chang-Gung Memorial Hospital, Chiayi, Taiwan; 25 Flagship Biosciences, Boulder, CO, USA; 26 Division of Pulmonary Sciences and Critical Care Medicine, Department of Medicine, University of Colorado Anschutz Medical Campus, Aurora, CO, USA; 27 Veterans Affairs Eastern Colorado Healthcare System, Aurora, CO, USA; 28 Department of Pathology, University of Colorado Anschutz Medical Campus, Aurora, CO, USA; 29 SAGA Diagnostics, Lund, Sweden; 30 Division of Oncology, Department of Clinical Sciences, Lund University, Lund, Sweden; 31 Early Cancer Detection Consultant, Bethesda, MD, USA; 32 Comprehensive Cancer Centre, King's College London, London, UK; 33 Division of Genetics and Epidemiology, Institute of Cancer Research, London, UK; 34 David H. Koch Institute for Integrative Cancer Research, Cambridge, MA, USA; 35 Department of Biology, Massachusetts Institute of Technology, Cambridge, MA, USA; 36 Cancer Research UK and UCL Cancer Trials Centre, London, UK; 37 Department of Oncology, University College London Hospitals, London, UK

## Abstract

A complete understanding of how exposure to environmental substances promotes cancer formation is lacking. More than 70 years ago, tumorigenesis was proposed to occur in a two-step process: an initiating step that induces mutations in healthy cells, followed by a promoter step that triggers cancer development^1^. Here we propose that environmental particulate matter measuring ≤2.5 μm (PM_2.5_), known to be associated with lung cancer risk, promotes lung cancer by acting on cells that harbour pre-existing oncogenic mutations in healthy lung tissue. Focusing on EGFR-driven lung cancer, which is more common in never-smokers or light smokers, we found a significant association between PM_2.5_ levels and the incidence of lung cancer for 32,957 EGFR driven lung cancer cases in four within-country cohorts. Functional mouse models revealed that air pollutants cause an influx of macrophages into the lung and release of interleukin-1β. This process results in a progenitor-like cell state within EGFR mutant lung alveolar type II epithelial cells that fuels tumorigenesis. Ultradeep mutational profiling of histologically normal lung tissue from 295 individuals across 3 clinical cohorts revealed oncogenic EGFR and KRAS driver mutations in 18% and 53% of healthy tissue samples, respectively. These findings collectively support a tumour promoting role for PM_2.5_ air pollutants and provide impetus for public health policy initiatives to address air pollution to reduce disease burden.

Barrier organs such as the lung are directly affected by exposure to environmental challenges. Accordingly, more than 20 environmental and occupational agents are lung carcinogens^2^, and exposure to these are of particular relevance in understanding lung cancer in the never-smoking population. Lung cancer in never-smokers (LCINS) is the eighth most common cause of cancer death in the UK and has distinct clinical and molecular characteristics compared with lung cancer in smokers^3^. LCINS frequently harbour adenocarcinomas with oncogenic EGFR mutations and are more commonly observed in female individuals and in individuals with East Asian ancestry compared with patients with Western ancestry^4^. Several factors have been proposed to explain the observed sex and geographical disparities of lung cancer driven by EGFR mutations, including germline genetics^5^, ethnicity, radon exposure, occupational carcinogen exposure and air pollution^6^. Air pollution accounts for 7 million deaths per year, with 99% of people living in areas that exceed World Health Organization guidelines (<5 μg m^−3^ annually)^7^. Particulate matter (PM) is a key constituent of air pollution and is classified by aerodynamic size. Fine particles ≤2.5 μm (PM_2.5_) are able to travel deep into the lung and are linked to multiple adverse health effects, including heart disease and lung cancer^7^. Traditionally, it is thought that carcinogens cause tumours by directly inducing DNA damage. However, recent data suggest that many carcinogens do not cause a detectable DNA mutational signature in tumours following exposure^8,9^. Genetic analyses of oesophageal cancer showed that mutational signatures do not fully explain the varied geographical incidence of this cancer^10^, and efforts that have profiled tumour genomes in LCINS failed to detect a dominant carcinogenic signal of mutations deriving from exogenous sources^11–13^. We propose that air pollutants might promote inflammatory changes in the lung tissue microenvironment that permit pre-existing mutated clones to expand, consistent with the two-stage carcinogenesis model of initiation and promotion^1^. To address this hypothesis, we combined epidemiological evidence with functional preclinical models and clinical cohorts to decipher potential mechanisms of air-pollution-induced lung tumour promotion and actionable targets for molecular cancer prevention (Extended Data Fig. 1a).

## Lung cancer incidence and PM_2.5_ levels

**Extended Data Figure 1:**
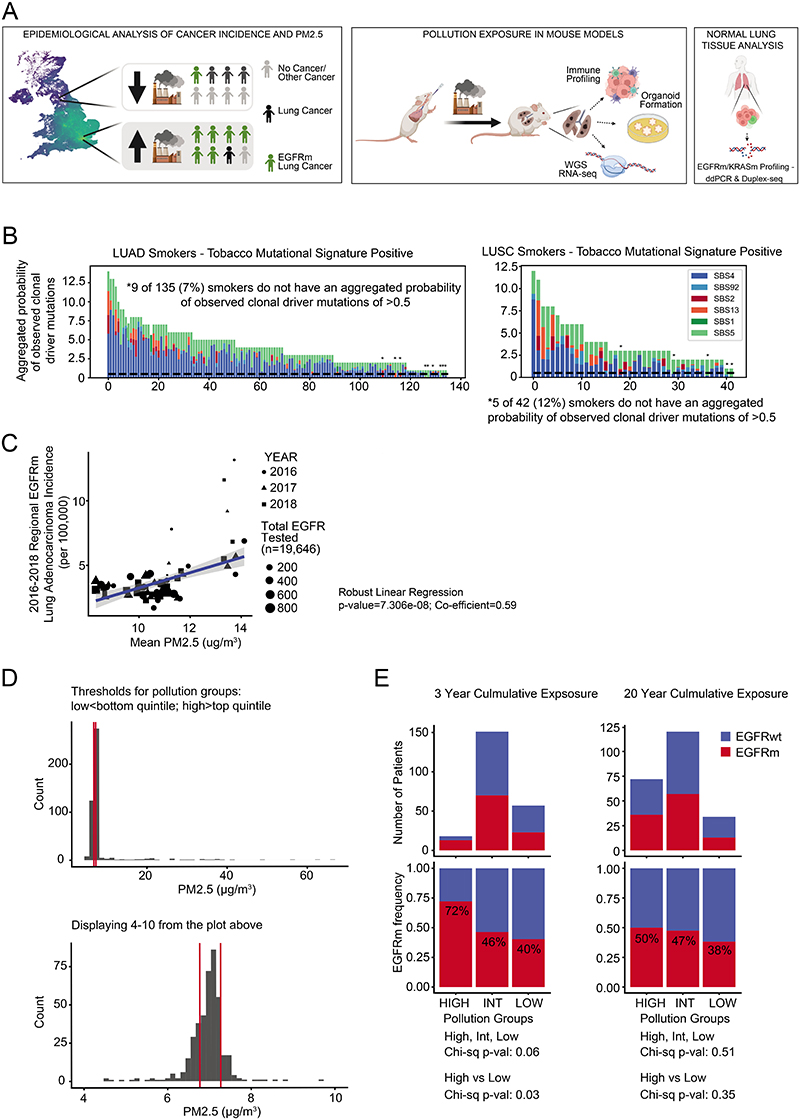
A) Study design schematic featuring the 3 aspects of the paper. LEFT: Epidemiological analysis of cancer incidence and PM_2.5_. MIDDLE: Pollution exposure in mouse models. RIGHT: Normal lung tissue analysis. B) TX421 Tumours from Smokers. Barplots indicating proportion of SNVs in each tumour attributed to each SBS mutational signature. The barplots (Top: Lung adenocarcinoma (LUAD), Bottom: Lung sqaumous cell carcinoma (LUSC)) reflect the probability that clonal driver mutations in patients, where smoking-related signatures have been detected, are caused by different mutational processes (SBS4 and SBS92 smoking, SBS2 and SBS13 APOBEC, SBS1 and SBS5 ageing). Each observed driver mutation in each patient is given a mutational-signature-causing probability based on the trinucleotide context and the signatures exposure of the patient (see Methods) and then these probabilities are aggregated. Asterisks represent patients where the smoking-related aggregated probabilities are below 0.5. C) Correlation between PM_2.5_ levels and *EGFR* mutant (EGFRm) adenocarcinoma lung cancer incidence in England. The blue line: robust linear regression line; grey shading: 95% confidence interval. D-E) The Canadian Lung Cancer Cohort. D) Distribution of 3 year and 20 year cumulative PM_2.5_ exposure levels for all patients in the Canadian cohort. Red lines mark the thresholds that were used to determine Low, Intermediate and High groups that are used in (D). These are the 1st (6.77ug/m^3^) and 5th quintiles (7.27ug/m^3^) of the distribution. The full distribution is displayed in the top plot, while the bottom plot displays a narrower range of 4-10 ug/m^3^ (for clarity). E) Counts and frequencies of EGFRm in the Canadian Cohort, where 3 year and 20 year cumulative PM_2.5_ exposure levels were available. Patients are grouped into high, intermediate and low groups based on thresholds established as described in (D). These groups are defined based on 3 year cumulative PM_2.5_ exposure data (left) and based on 20 year cumulative PM_2.5_ exposure data (right). The bar plots display the counts and frequency of EGFRm amongst patients within each group

**Fig. 1:**
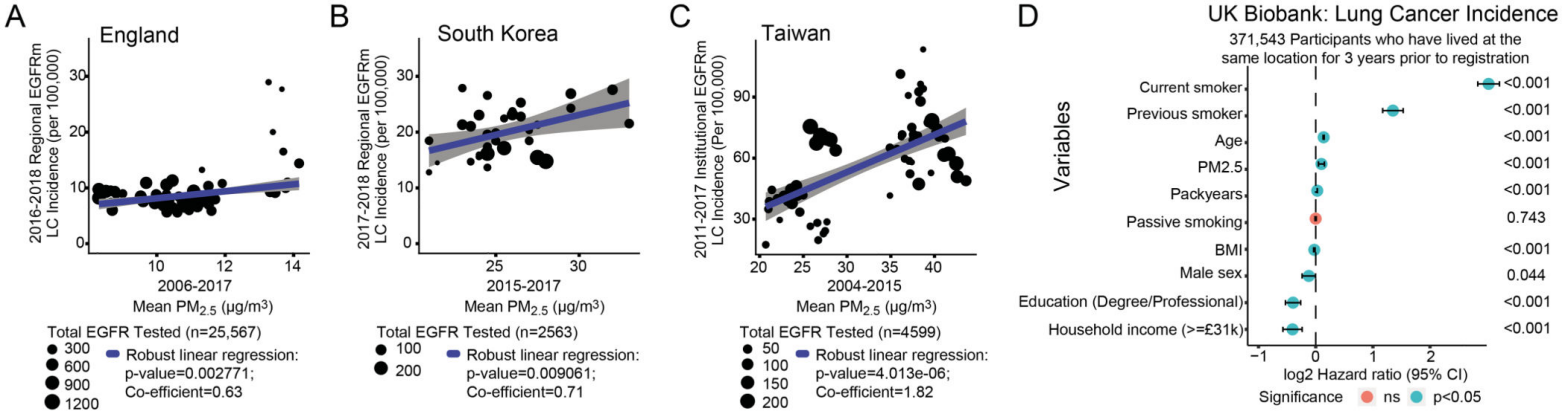
Exploring the association between cancer and air pollution. a–c, Scatter plots showing relationships between PM_2.5_ levels and estimated EGFR-driven (EGFR mutant; EGFRm) lung cancer (LC) incidence (per 100,000 population) at the country level in England (a), South Korea (b) and Taiwan (c). Grey shading indicates 95% confidence intervals. d, Forest plot indicating the relationship between lung cancer risk and various co-variates, including residential PM_2.5_ exposure levels (range: 8.17–21.31 μg m^−3^) in the UK Biobank dataset. Only participants who have lived at the same location for 3 years before registration (n = 371,543) are included. Each co-variate is displayed on a different row. Cox regression P values are indicated on the right. BMI, body–mass index; NS, not significant

In a companion article^14^, our analysis of lung adenocarcinoma (LUAD) tumours from the TRACERx 421 cohort revealed that despite a history of smoking, a minority of patients (8%) lacked evidence of smoking-mediated mutagenesis, including 6.4% of patients with >15 years of smoking. Consistent with that analysis, in the current study, 7–12% of smokers in the TRACERx 421 cohort did not have a driver single nucleotide variant that could be attributed to a smoking-related single base substitution (SBS) mutation signature (SBS4 or SBS92) (Extended Data Fig. 1b). This result suggests that smoking may promote cancer through additional mechanisms^15^. To understand whether air pollutants can promote the formation of lung tumours without inducing exogenous mutational signatures, we studied EGFR-driven lung cancer, which has a high prevalence in LCINS (in England, the probability that a LCINS is caused by an EGFR-driven tumour is 36–40%), owing to its low mutational burden^11–13^ and greater incidence in countries in Asia^4^ (Supplementary Tables 1–3). To examine the relationship between air pollutants and EGFR-driven lung cancer incidence, we used several ecological correlation analyses, acknowledging that these analyses only provide estimates of incidence. We considered data from three countries to explore different ranges of PM_2.5_ air pollution and ethnicities: England (92% white; PM_2.5_ interquartile range (IQR): 9.95–11.2 μg m^−3^); South Korea (>99% Asian^16^; PM_2.5_ IQR: 24.0–27.0 μg m^−3^); and Taiwan (>98% Asian^17^; PM_2.5_ IQR: 24.3–38.2 μg m^−3^) (Supplemetary Tables 1–3). In each country, there was a consistent relationship between PM_2.5_ levels (average concentration per geographical area) and estimated EGFR-driven lung cancer incidence (Fig. 1a–c). The relative rates of EGFR-driven lung cancer incidence (per 100,000 population), per 1 μg m^−3^ increment of PM_2.5_ levels were 0.63 (P = 0.0028) in England, 0.71 (P = 0.0091) in South Korea and 1.82 (P = 4.01 ×10^−6^) in Taiwan. When restricting the English cohort to adenocarcinoma cases, the relationship remained significant (Extended Data Fig. 1c). In the above analyses, we were not able to account for the migration of individuals before the diagnosis of lung cancer. As such, we analysed samples from a group of female patients with LCINS (92% LUAD, n = 228) from British Columbia, Canada. For this dataset, PM_2.5_ cumulative exposure was individually calculated for each individual through a detailed residential history from birth to current address. Most of the patients in this group (83%) were born outside Canada, and 46.7% harboured an EGFR mutation. An analysis of 3-year and 20-year PM_2.5_ cumulative exposure (Methods) revealed that the frequency of EGFR-driven lung cancer cases was significantly higher after 3 years of high air pollutant exposure compared with low exposure (EGFR mutation frequency in high compared with low pollution (Methods): 73% versus 40%, respectively, P = 0.03; Extended Data Fig. 1d,e). Of note, this association was not observed after 20 years of high compared with low cumulative exposure (high versus low pollution: 50% versus 38%, respectively, P = 0.35; Extended Data Fig. 1d,e). This result could indicate that 3 years of high PM_2.5_ exposure may be sufficient for EGFR-driven lung cancers to arise. To explore whether 3 years of cumulative PM_2.5_ exposure is associated with lung cancer in an independent cohort not restricted to EGFR-driven cases, we obtained data from 407,509 participants in the UK Biobank. An analysis that included all participants, regardless of changes in residential location before registration, demonstrated that PM_2.5_ levels (calculated at 1 μg m^3^ increments) were associated with lung cancer incidence (hazard ratio (HR) = 1.08 (95% confidence interval: 1.04–1.12), raw P ≤ 0.001, false discovery rate (FDR) = 0.001; Supplementary Table 4), a result that is consistent with a previous analysis^18^. By contrast, lung cancer incidence was not associated with outdoor radon levels (HR = 0.96 (0.89–1.03), P = 0.262; Methods). Interaction tests between ever-smoking status and PM_2.5_ exposure levels suggested that smoking and high PM_2.5_ levels may have a combined effect on lung cancer risk (P = 0.049; Supplementary Table 4). We also noted nominal significance (raw P < 0.05, FDR > 0.05) for lip and oropharyngeal cancer (HR = 1.10 (1.01–1.19), raw P = 0.023, FDR = 0.215) and mesothelioma (HR = 1.11 (1.00–1.24), raw P = 0.048, FDR = 0.339; Supplementary Table 4 and Methods). Finally, we restricted our analysis to participants resident at the same address in the 3 years before registration (n = 371,543). This analysis showed that the relationship between lung cancer incidence and PM_2.5_ exposure levels remained significant (Fig. 1d; HR = 1.07 (1.03–1.11); P ≤ 0.001). Collectively, these data, combined with published evidence^6^, indicate that there is an association between the estimated incidence of EGFR-driven lung cancer and of PM_2.5_ exposure levels and that 3 years of air pollution exposure may be sufficient for this association to manifest.

## PM-mediated promotion of lung cancer

**Extended Data Figure 2:**
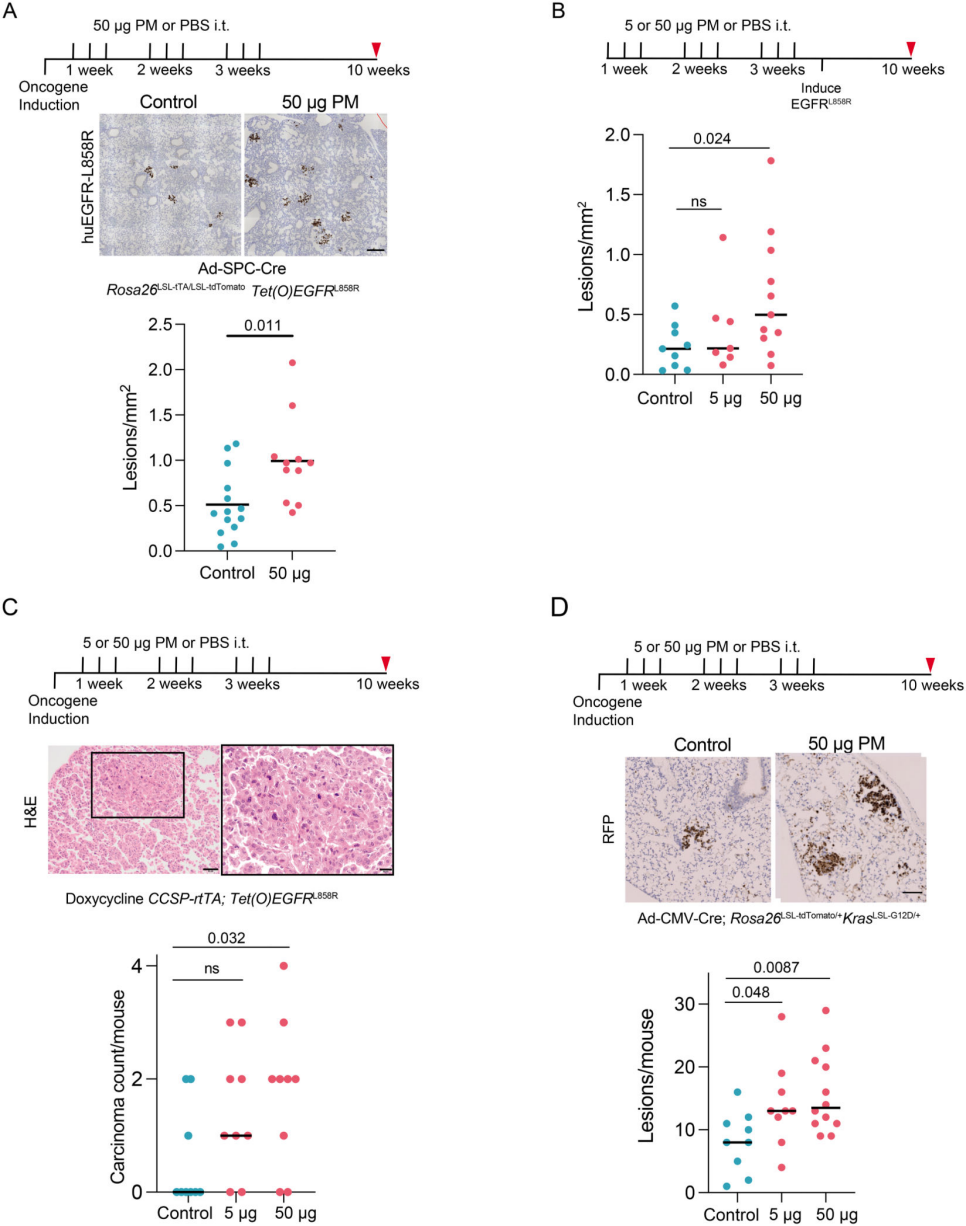
A) Schematic of PM exposure and representative IHC of ET mice induced with AT2-specific Ad5-SPC-Cre exposed to PM or PBS control and quantification of neoplastic lesions (n=14 PBS, n=11 PM). Mann-Whitney test. B) Schematic of PM exposure followed by expression of *EGFR*^L858R^ and quantification of precancerous lesions/mm^2^ of lung tissue (n=9 PBS; n=7 5 μg; n=11 50 μg PM). One-way ANOVA. C) Schematic of PM exposure and representative H&E of a lung adenocarcinoma in a 50 μg PM exposed, doxycycline treated *CCSP-rtTa; TetO-EGFR^858R^* mice; quantification of number of adenocarcinomas per mouse below (n = 9 per group). One-way ANOVA. D) Schematic of PM exposure and representative IHC for red fluorescent protein (RFP, marks tdTomato+ cells) in *Rosa26*^LSL-tdTomato/+^;*Kras*^LSL-G12D/+^ mouse model in control or 50 μg PM exposed conditions; quantification of number of hyperplastic lesions per mouse (n= 9 control, n=9 5 μg and n=12 50 μg). One-way ANOVA. Scale bar 50 μm (C main, H), 20 μm (C insert), 100 μm A & D

**Fig. 2:**
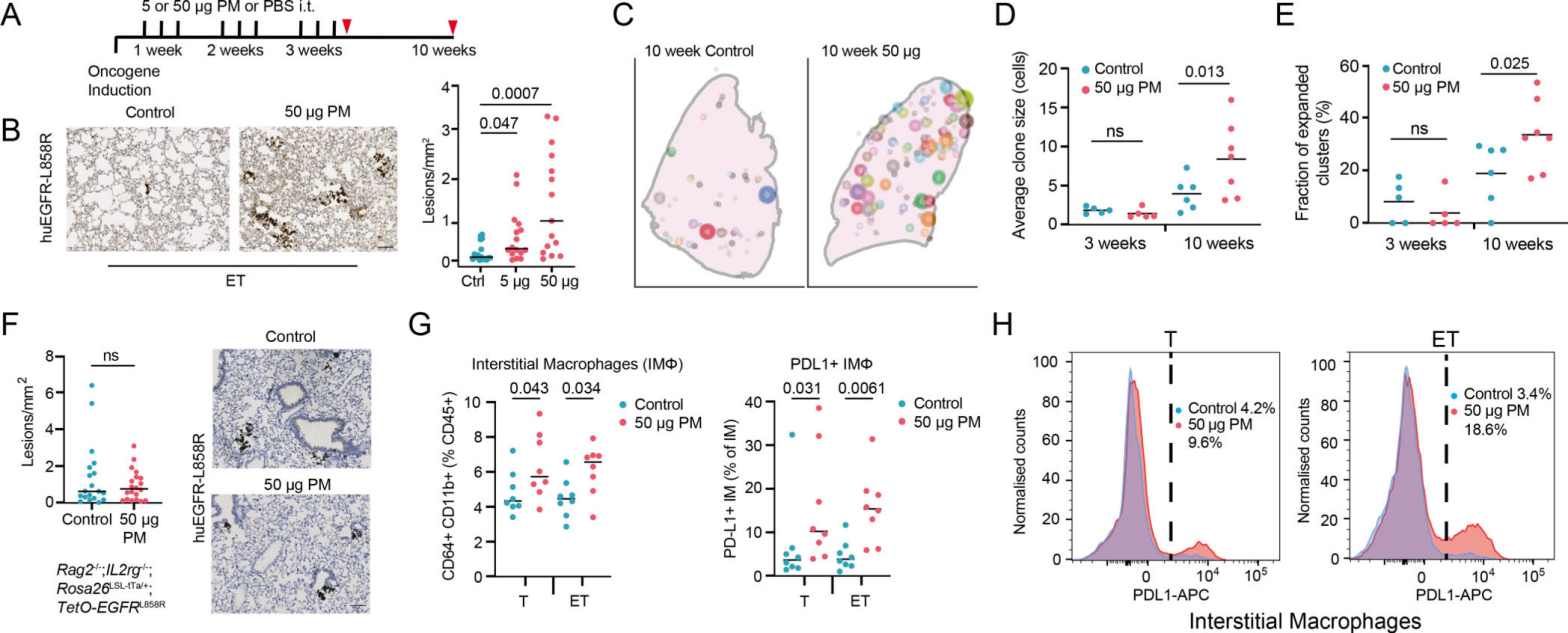
PM promotes lung tumorigenesis. a, Schematic of the experiment. Induction of the oncogene was followed by exposure (black lines) through intratracheal (i.t.) administration of PM or PBS (control). Timing of tissue collection is indicated by the red triangles. b, Left, representative immunohistochemistry (IHC) images of human EGFR^L858R^ in ET mice exposed to PBS or PM at 10 weeks. Right, quantification of human EGFR^L858R^-positive neoplasia per mm^2^ of lung tissue (n = 16 for the PBS and 5 μg PM groups, n = 15 for the 50 μg PM group). One-way analysis of variance (ANOVA). c, Representative diagram of spatially segmented human EGFR^L858R^-positive clusters in lung lobes, with the size of clusters proportional to EGFR^L858R^ cell number at 10 weeks. d,e, Quantification of average cluster size (d) and fraction of expanded clusters (>5 cells) (e) in mice exposed to PM or PBS over time (n = 5 for 3 week control and 50 μg PM; n = 6, 10 week control; n = 7, 10 week 50 μg PM). One-way ANOVA. f, Left, quantification of lesions in Rag2^−/−^;Il2rg^−/−^;Rosa26^LSL-tTa/+^;TetO-EGFR^L858R^ mice at 10 weeks after EGFR induction. Right, representative IHC images of EGFR^L858R^ (n = 19 for control, n =20 for 50 μg PM). Mann–Whitney test. g, Proportion of interstitial macrophages (IMs) and PD-L1+IMs within lung tissue in Rosa26^LSL-tdTomato/+^ mice and ET mice determined by flow cytometry 24 h after final PBS (control) or PM exposure (n = 8 per group). One-way ANOVA. h, Representative histogram showing PD-L1 expression within lung IMs in Rosa26^LSL-tdTomato/+^ (left) and ET (right) mice exposed to control or PM conditions. Scale bar, 100 .m (b, f). Specific P values are indicated on the charts

Next we used genetically engineered mouse models of LUAD to functionally examine whether PM exposure promotes lung tumour development. We induced expression of oncogenic human EGFR^L858R^ in mouse lung through the intratracheal delivery of adenoviral-CMV-Cre to mice engineered with Rosa26^LSL-tTa/LSL-tdTomato^;TetO-EGFR^L858R^ alleles (ET mice). Mice were exposed to physiologically relevant doses of fine PM or PBS for 3 weeks after the induction of EGFR^L858R^, and tumour burden was assessed 10 weeks after oncogene induction (Fig. 2a and Methods). In this model, rare, sporadic lung epithelial cells expressing oncogenic EGFR expanded to form pre-invasive neoplasia by 10 weeks (Fig. 2a,b). An analysis of ET mice at 10 weeks after exposure to PM revealed a dose-dependent increase in the number of pre-invasive neoplasias (PBS compared with 5 μg PM, P = 0.047; PBS compared with 50 μg PM, P = 0.0007; Fig. 2b). PM also enhanced the number of pre-invasive neoplasia when EGFR^L858R^ induction was restricted to alveolar type II (AT2) cells using lineage-specific adenoviral-SPC-Cre (Extended Data Fig. 2a). Exposure to PM before the CMV-Cre-mediated induction of EGFR^L858R^ also resulted in an increased number of early neoplasias (P = 0.024; Extended Data Fig. 2b), which indicated that PM exposure before or after oncogene induction is sufficient to promote carcinogenesis.

**Extended Data Figure 3:**
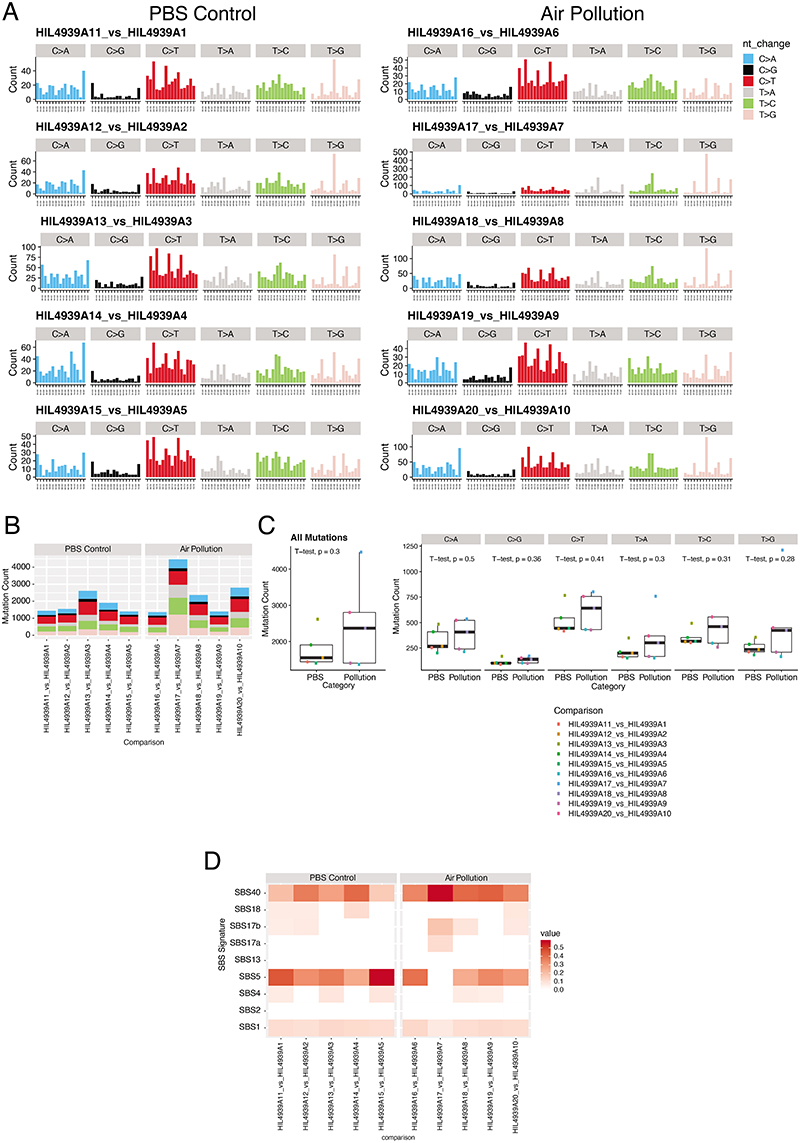
WGS analysis of tumours from ET mice exposed to air pollution (n=5) and those exposed to PBS controls (n=5). Each mouse tumour is compared vs the corresponding germline from the same mouse. A) Mutational profiles for each tumour sample according to the mutation trinucleotide context. LEFT: PBS Controls, RIGHT: 50 μg PM. B) Barplots indicate the counts of mutations in each sample, where bars are colored based on the base change. C) Boxplot comparing the counts of mutations between tumours from pollution exposed mice (50 μg PM) and tumours from PBS exposed mice (PBS Control). All mutations are summarised in one plot on the left, and are then further divided based on the base change of the mutation (n=5 mice per group). Two-sided T-test comparing numbers of mutations between PBS and Air Pollution p-values are displayed. The boxplot line represents the median, the hinges of the box represent the 1st and 3rd quartiles and the limits of the whiskers represent the 1.5 interquartile range. D) Attribution of mutations in each tumour sample to each single base substitution (SBS) mutation signature. The shading indicates the weight of the signature within each sample. Majority of the weights have been assigned to ageing related signatures (SBS40, SBS5, SBS1) Komogolomov-Smirnoff test p-value=0.26-0.68

**Extended Data Figure 4:**
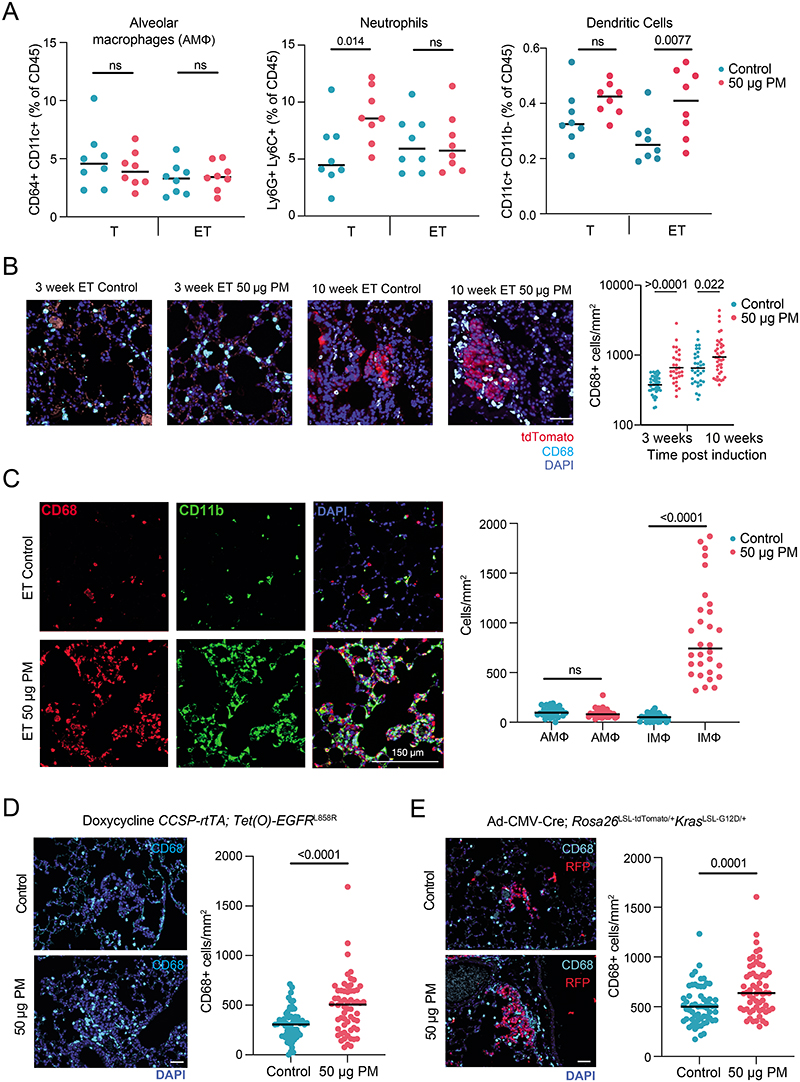
A) Immune cell frequencies in the lungs determined by flow cytometry 24 hours post-exposure from induced tdTomato (T) and *EGFR* mutant (ET) mice after 50 μg PM (red) or control (blue) (n=8 mice per group). Data are presented as the frequency among live CD45+ immune cells. One-way ANOVA. B) Representative immunofluorescent images of CD68+ macrophages (cyan) and tdTomato+ *EGFR* mutant cells (red) within ET lungs exposed to control or 50 μg PM. Quantification of CD68+ cells per mm^2^ of lung tissue (n= 4 mice per group). C) Representative immunofluorescent images of CD68 (red), CD11b (green) and merged images from induced ET mice after 3 weeks of exposure to PBS (top) or 50 μg PM (bottom). Quantification of alveolar macrophages (AMΦ, CD68+CD11b-) and interstitial macrophages (IMΦ, CD68+CD11b+) per mm^2^ of lung tissue, selecting 10 x random 500 μm^2^ fields of view per mouse (n= 3 mice per group). One-way ANOVA. D) Representative immunofluorescent images of CD68+ macrophages (cyan) within *CCSP-rtTA; TetO-EGFR^L858R^* lungs treated with PBS (top) or 50 μg PM (bottom) 10 weeks post oncogene induction; quantification of CD68+ cells per m^2^ of lung tissue, selecting 20 x random 500 μm^2^ fields of view per mouse (n= 3 mice per group). Unpaired t-test. E) Representative immunofluorescent images of CD68+ macrophages (cyan) and tdTomato+ *Kras^G12D^* mutant cells (red) within KT lungs treated with PBS (top panel) or 50 μg PM (bottom) 10 weeks post oncogene induction; quantification of CD68+ cells per mm^2^ of lung tissue, selecting 20 x 500 μm^2^ fields of view containing RFP+ cells per mouse (n= 3 mice per group). Unpaired t-test. Scale bar 50 μm B & D, 150 μm E. Gating strategies for flow cytometry analysis provided in Extended Data Figure 6. Statistical analysis by one-way ANOVA for B, D, E & G. Scale bars 100 μm (B,F,E)

PM exposure also increased the number of adenocarcinomas in a more aggressive CCSP-rtTa;TetO-EGFRL858R model of doxycycline-inducible LUAD (P = 0.032; Extended Data Fig. 2c). Moreover, the number of hyperplasias in an adenoviral–CMV-Cre KrasG12D model of lung cancer was also increased (Rosa26LSL-tdTomato/+;KrasLSL-G12D/+ mice; 5 μg PM, P = 0.048; 50 μg PM, P = 0.0087; Extended Data Fig. 2d). Together, these data suggest that PM can promote tumour progression in both oncogenic Kras and EGFR models of LUAD. Next we explored the mechanisms by which PM might promote EGFR-driven lung tumorigenesis. Spatial analysis of clonal dynamics throughout early tumorigenesis in ET mice indicated that the expansion of EGFR mutant cells did not occur during PM exposure but manifested in the period after PM cessation (3 weeks, P = 0.82; 10 weeks, P = 0.013; Fig. 2c,d and Methods). Both the fraction of EGFRL858R cells that grew into clusters and the number of cells within these clusters were increased in PM-exposed ET mice at 10 weeks but not at 3 weeks (Fig. 2d,e). These data suggest that PM acts in two ways to promote early tumorigenesis: by increasing the number of EGFR mutant cells with the potential to form a tumour and by increasing the proliferation rate of EGFR mutant cells within these early lesions. To test whether PM induces DNA mutagenesis, we performed whole-genome sequencing on tumours from ET mice exposed to PM or PBS. We did not observe an increase in the number of mutations in tumours from PM-exposed mice (P = 0.30; Extended Data Fig. 3a–c), or an enrichment in any established SBS signatures (P = 0.26–0.68). This result suggests that short-term exposure to PM does not enhance mutagenesis. Most of the mutations in tumours from PM-exposed mice and PBS-treated mice were attributable to the ageing SBS signatures (Extended Data Fig. 3d). We next examined whether the immune system is required for PM-enhanced EGFR-driven tumorigenesis. We crossed Rosa26LSL-tTa;TetO-EGFRL858R mice with Rag2^−/−^;Il2rg−/− mice, which lack T cells, B cells and natural killer cells and have an altered myeloid compartment^19^, to generate immune-deficient EGFR mutant mice after Cre delivery (Rag2^−/−^;Il2rg^−/−^;Rosa26^LSL-tTa/+^;TetO-EGFR^L858R^). In contrast to the ET mice (Fig. 2b), exposure to PM following EGFR^L858R^ induction in these immune-deficient mice did not result in increased neoplasia. This result suggests that a competent immune system is required for PM-enhanced EGFR-driven lung tumorigenesis (P = 0.879; Fig. 2f). The inhalation of toxic particles induces a local response in the lung, which is mediated by macrophages and lung epithelial cells^20^. We profiled the acute myeloid response to PM in immunocompetent lungs from Rosa26^LSL-tdTomato/+^ mice (control) and from mice harbouring an EGFR mutation (ET mice) 24 h after the final exposure to PM. We observed an increase in the proportion of interstitial macrophages (control mice, P = 0.043; ET mice, P = 0.034; Fig. 2g) and an increase in PD-L1 expression on these cells in both control and ET mice following PM exposure (control mice, P = 0.031; ET mice, P = 0.0061; Fig. 2g,h). No change was observed in alveolar macrophages (Extended Data Fig. 4a). In addition, lungs from control mice displayed an increase in neutrophils, whereas dendritic cells were increased in lungs from ET mice (Extended Data Fig. 4a). Immunofluorescence staining of lungs from ET mice with the pan-macrophage marker CD68 revealed a greater density of macrophages after PM exposure, both acutely (24 h after exposure) and at 7 weeks after exposure (3 weeks, P ≤ 0.0001; 10 weeks, P = 0.022; Extended Data Fig. 4b). These macrophages were confirmed to be CD11b^+^CD68^+^ interstitial macrophages (Extended Data Fig. 4c). We also observed an increased number of macrophages in both the doxycycline-inducible EGFR^L858R^ mice and Rosa26^LSL-tdTomato/+^; Kras^LSL-G12D/+^ mice 10 weeks after induction and PM exposure (Extended Data Fig. 4d,e). These data support the hypothesis that transient PM exposure is associated with enhanced and sustained lung macrophage infiltration beyond the period of PM exposure.

## PM-mediated AT2 cell reprogramming

**Extended Data Figure 5:**
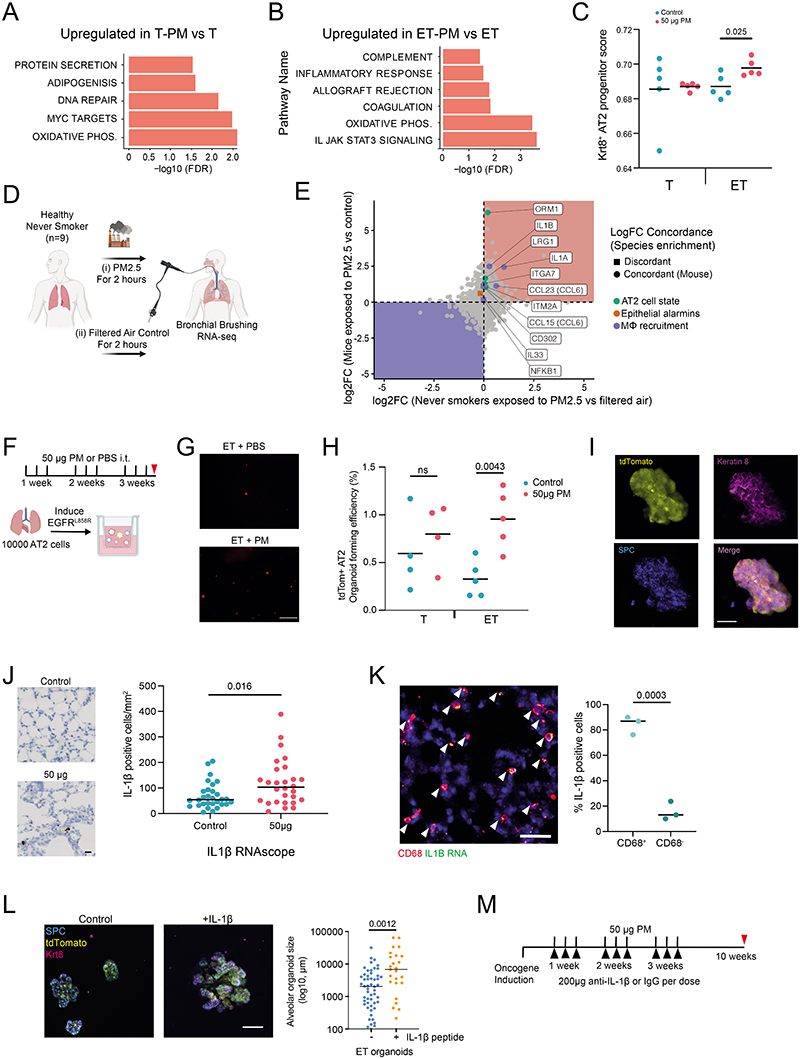
A-B) Significantly enriched GSEA pathways upregulated in T-PM lung epithelial cells compared to T control mice (A), in ET-PM lung epithelial cells compared to ET control mice (B). For each comparison, barplots indicate the -log10(FDR) of the Komogolomov-Smirnoff test p-value for each pathway. C) Krt8+ AT2 progenitor score derived from scRNAseq of bleomycin treated mouse lung used to deconvolute bulk RNA-seq of T and ET mice exposed to 50 μg PM or PBS, (n= 5 mice per group). Welch's t-test between control and PM. D) Schematic displaying experimental set-up of clinical exposure study in never-smoker volunteers, crossover design with (i) and (ii) in random order separated by 4-week washout. E) Fold change (FC) of significantly upregulated genes (identified in mouse) compared to the fold change of genes changed in the clinical exposure study. Common directionality across species indicated by colour (negative: blue background; positive: red background). F) Schematic of AT2 culture from T or ET mice exposed to 50 μg PM or PBS, with induction of tdTomato or oncogene *ex vivo*. G) Representative fluorescent images of tdTomato+ AT2 organoids at day 14 from ET mice exposed to PBS or 50 μg PM in vivo. Scale bar 100 μm. H) Quantification of tdTom+ AT2 organoid forming efficiency, data represents averages from 2 technical replicates/mouse; n=4 mice from T control and PM; n=5 mice for ET control and PM. One-way ANOVA. I) Representative fluorescent imaging of tdTomato (yellow), Keratin 8 (magenta), SPC (blue) on a wholemount AT2 organoid from an ET mouse treated with 50 μg PM. Scale bar is 20 μm. J) LEFT: Representative IL-1β RNAscope performed on lungs from ET mice treated with PBS or 50 μg PM after 3 weeks of exposure. Scale bar 20 μm. RIGHT: Quantification of IL-1β+ cells per mm^2^ of lung tissue from 30 random fields of view (control, n = 3 mice) and 28 fields of view (50 μg PM, n = 3 mice). Mann-Whitney test p-value is displayed. K) LEFT: Representative image of IL-1β RNAscope (green) in CD68 positive (red) macrophages, arrows indicate positive macrophages. n=3 mice per group. Scale bar 50 μm. RIGHT: Quantification of IL-1β positive CD68+ cells at 3 weeks post induction in ET mice following exposure to PM. Mann-Whitney test. L) LEFT: Representative fluorescent images of *EGFR*^L858R^ naive (non-PM exposed) AT2 organoids from ET mice treated with control or IL-1β *in vitro*. tdTomato (yellow) organoids stained with SPC (blue) and Keratin 8 (magenta). Scale bar 50 μm. RIGHT: Quantification of organoid size with each dot representing an organoid at day 14 of control (blue) or IL-1β treated (orange). Organoids derived from n=2 mice per group. Mann-Whitney test. M) Schematic of anti-IL-1β treatment treatment (black triangles) during PM exposure (black lines) and harvest (red triangle)

**Fig. 3:**
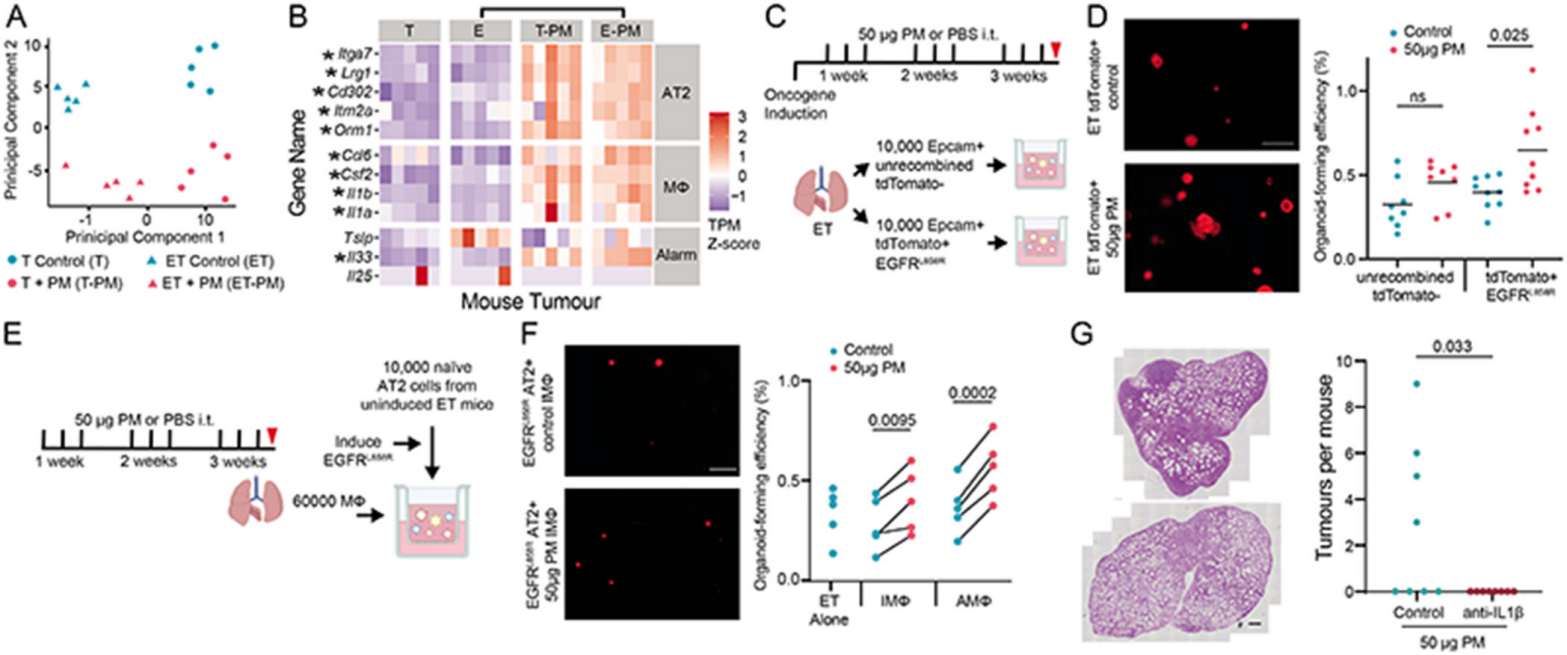
Increased progenitor-like ability of EGFR mutant cells following PM exposure. a, PC analysis plot of RNA-seq data from epithelia samples taken from recombined Rosa26^LSL-tdTomato/+^ mice (T) and ET mice exposed to 50 μg PM or PBS. b, Heatmap of progenitor AT2 cell state (AT2), macrophage recruitment and epithelial alarmin (Alarm) gene expression in all mouse tumour samples. The colour scale in the heatmap represents high (red) to low (blue) transcript per million (TPM) expression z-scores. Asterisks indicate significantly different (FDR < 0.05) gene expression between ET and ET + PM (Methods). c, Schematic of the epithelial organoid assay. Lungs were taken from mice exposed to PM or PBS, followed by isolation and culture of epithelial (positive for epithelial cellular adhesion molecule (EpCAM^+^)) cells. d, Left, representative fluorescent images of tdTomato^+^ organoids at day 14 from ET mice exposed to PBS (control) or PM in vivo. Right, OFE within unrecombined (tdTomato^−^) or recombined (tdTomato^+^) EpCAM^+^ lung cells from ET mice exposed to PBS or PM. Two mice were pooled for each biological replicate for sufficient tdTomato^+^ cells. Data represent mean from tdTomato^-^, n = 8 (16 mice); tdTomato^+^EGFR^L858R^, n = 9 (18 mice). One-way ANOVA. e, Schematic of macrophage isolation from mice exposed to PM or PBS and co-cultured with naive (non-PM exposed) EGFR^L858R^ AT2 cells. f, Left, representative fluorescent images of tdTomato^+^EGFR^L858R^ AT2-cell-derived organoids from ET mice, co-cultured with IMs exposed to PM or PBS. Right, quantification of OFE of EGFR^L858R^ AT2 cells alone and compared with AT2 cells from the same mouse co-cultured with alveolar macrophages (AMs) exposed to PBS or PM (n = 5 mice, data are average of 2 technical replicates per mouse). Paired-t test. g, Left, representative haematoxylin and eosin images of PM-exposed mice treated with IgG control antibody or anti-IL-1β throughout exposure duration. Right, quantification of tumours (n = 8 mice per group). Mann–Whitney test. Scale bar, 500 μm (d,f). The illustrations in c and e were created using BioRender (https://biorender.com)

To investigate the effects of PM exposure on early tumorigenesis, lung epithelial cells were purified by flow cytometry, and RNA sequencing (RNA-seq) was performed acutely following exposure in four different conditions: control mice exposed to PM or to PBS, and ET mice exposed to PM or to PBS. Principal component (PC) analysis of gene expression values showed that PM exposure accounted for 19% of the variance (genes differentially expressed between control mice that were exposed to PM and control mice that were exposed to PBS display higher PC2 ranks, P < 0.001) and EGFR mutation accounted for 38% of the variance (genes differentially expressed between ET mice that were exposed to PM and control mice that were exposed to PM display higher PC1 ranks, P < 0.001; Fig. 3a and Supplementary Table 5). Gene set enrichment analysis of PM-treated ET mice showed that compared with PBS-treated ET mice, the IL-6–JAKS–TAT pathway, inflammatory responses and the allograft rejection pathway were only upregulated following PM exposure in epithelium with EGFR mutations. This was in contrast to the pathways induced by PM exposure in control mice (Extended Data Fig. 5a,b). In ET mice, PM exposure led to an upregulation of genes known to regulate macrophage recruitment, including those that encode interleukin-1β (IL-1β), GM-CSF, CCL6 and NF-κB and the epithelial-derived alarmin IL-33 (Fig. 3b). AT2 cells are a probable cell of origin of lung adenocarcinoma^21^, and the bleomycin lung injury model has identified a keratin 8-positive (KRT8^+^) subset of these cells as progenitors that mediate alveolar regeneration driven by inflammatory signals such as IL-1β^22^. Consistent with our data showing that PM can promote tumorigenesis in EGFR^L858R^ AT2 cells, we noted upregulation of genes previously associated with AT2 progenitor cell states (Fig. 3b). Deconvolution of our bulk RNA-seq expression data with signals trained on a single-cell RNA-seq dataset of bleomycin-treated mouse lungs^23^ identified an increased AT2 activated progenitor score only in ET mice exposed to PM (Extended Data Fig. 5c and Methods). This result suggests that EGFR^L858R^ AT2 cells are transcriptionally reprogrammed to this progenitor cell state following PM exposure. We compared the mouse RNA-seq data to a human clinical crossover study in which lung brushings from individuals who never smoked were taken after exposure to diesel exhaust and filtered air^24^,^25^ (Extended Data Fig. 5d). A number of significantly upregulated genes within the mouse lung epithelium were also upregulated in human lung epithelium (but not reaching significance) after PM exposure, including markers of macrophage recruitment (IL1B and IL1A) and markers of AT2 progenitor state (ORM1 and LRG1) (Extended Data Fig. 5e and Supplementary Table 5). These results identify PM-induced transcriptional changes in lung epithelium associated with inflammation and lung progenitor cell states^22^. To test whether these alterations are reflected in functional differences in epithelial cell progenitor behaviour, we isolated lung epithelial cells from ET mice following in vivo exposure to PM, and cultured them in a 3D lung-organoid-formation assay with lung fibroblasts^26^ ex vivo (Fig. 3c). Non-recombined (EGFR wild-type) cells from ET mice exposed to PM did not display an increase in organoid-formation efficiency (OFE; P = 0.075; Fig. 3d). By contrast, recombined tdTomato^+^EGFR^L858R^ cells demonstrated an increase in OFE (P = 0.025; Fig. 3d). To validate whether AT2 cells specifically are functionally altered by PM, we purified AT2 cells from non-induced ET mice and control mice exposed to PM, induced recombination in vitro^27^ and then plated the cells (Extended Data Fig. 5f). Increased OFE was observed only in tdTomato^+^EGFR^L858R^ AT2 cells from mice exposed to PM in vivo (P = 0.0043; Extended Data Fig. 5g,h). This result is consistent with our in vivo data (Extended Data Fig. 2a,b) and demonstrates that reversing the temporal order of oncogenic mutation initiation and PM exposure also increases OFE. PM-exposed AT2 organoids were KRT8^+^ and SPC^+^, consistent with an AT2 progenitor state (Extended Data Fig. 5i). These data suggest that the combination of in vivo exposure to PM and induction of the EGFR^L858R^-driver mutation increases AT2 cell progenitor function, a phenotype that is not seen with PM exposure or expression of EGFR^L858R^ alone.

## PM induces IL-1β production from macrophages

We proposed that lung macrophages, which release inflammatory cytokines when exposed to PM^28^, may be central to tumour promotion. We isolated AT2 cells from ET mice not exposed to PM, induced EGFR^L858R^ expression ex vivo and co-cultured the cells with macrophages exposed in vivo to either PM or PBS (Fig. 3e). Both PM-exposed interstitial macrophages and alveolar macrophages increased the OFE of EGFR mutant AT2 cells (interstitial, P = 0.0095; alveolar, P = 0.0002; Fig. 3f). This result indicates that a key mediator of PM-induced inflammation arises from macrophages. Previous reports have shown that IL-1β, derived from lung macrophages, is required for the formation of KRT8^+^ AT2 progenitor cells after bleomycin injury^22^. Therefore, we reasoned that IL-1β may be a key molecular mediator of tumour promotion and the pollutant-driven change in cell state. IL-1β was upregulated in PM-treated lungs and predominantly appeared within CD68^+^ macrophages (Extended Data Fig. 5j,k). Furthermore, treatment of EGFR mutant AT2 cells in vitro with IL-1β resulted in larger KRT8^+^SPC^+^ organoids (Extended Data Fig. 5l). Finally, to test the requirement of IL-1β in PM-enhanced adenocarcinoma formation, we initiated oncogene expression in the doxycycline-inducible CCSP-rtTa;TetO-EGFR^L858R^ model and exposed mice to PM with concomitant administration of an anti-IL-1β or a control antibody (200 μg per dose; Extended Data Fig. 5m). Treatment with an anti-IL-1β antibody during PM exposure was sufficient to attenuate EGFR-driven LUAD formation (P = 0.034; Fig. 3g). Collectively, these data establish that PM-exposed macrophages are sufficient to induce a progenitor-like state in EGFR mutant AT2 cells. Moreover, macrophages are a key source of IL-1β in response to PM and IL-1β signalling is required for the promotion of PM-mediated EGFR-driven LUAD.

## Oncogenic mutations in healthy lung

**Fig. 4:**
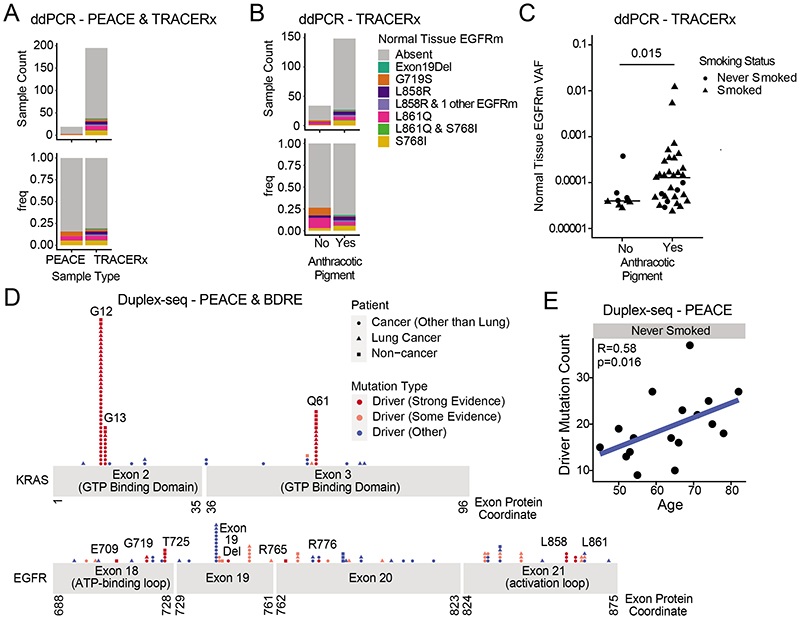
Mutational landscapes of healthy lung tissue. a, Counts and proportions of non-cancerous lung samples from PEACE (n = 19) and TRACERx (n = 195) patients that harbour EGFR mutations (EGFRm) identified using ddPCR. The EGFR mutation type is indicated by the colour of the bars (key in b). b, Count and proportion of healthy lung samples from the TRACERx dataset (organized according to anthracotic pigment content: yes (n = 149); no (n = 34)) that harbour EGFR mutations identified by ddPCR. The EGFR mutation type is indicated by the colour of the bars. c, Proportion test Beeswarm plot of ddPCR TRACERx data indicating the VAFs of EGFR mutations. Samples organized according to presence (yes; n = 31) or absence (no; n = 9) of anthracotic pigment. Shapes of dots indicate smoking status. Two-sided t-test. d, Gene models of KRAS (top) and EGFR (bottom), where dots represent mutations identified in the Duplex-seq PEACE and Duplex-seq BDRE cohorts. The position of the dots correspond to the loci of the mutations, whereas the height of the stack indicates the count of the number of mutations at a particular protein coordinate. The shape of the dot indicates the disease diagnosis of the patient, whereas the colour of the dot indicates the mutation type. e, Scatter plot displaying the correlation between age and the number of driver mutations identified in samples from never-smoker individuals (n = 17) in the Duplex-seq PEACE cohort, for which the panel comprised genomic loci in 31 genes, including EGFR and KRAS. Spearman correlation coefficient and P value are indicated in the plot

The model of tumour initiation and promotion is contingent on histologically normal tissue cells harbouring oncogenic driver mutations^1^. In 15 reported studies involving deep sequencing of human histologically normal tissues from different anatomical sites (n = 9,380 samples from 380 patients), an oncogenic EGFR^L858R^ mutation was only reported in a single clone from a skin microbiopsy, which indicated that these mutations are rare (Supplementary Table 6). Using digital droplet PCR (ddPCR) and duplex sequencing (Duplex-seq), we sought for evidence of EGFR-driver mutations in non-cancerous lung tissue from patients with lung cancer or with cancers of other organs and from individuals with no evidence of cancer (Extended Data Figs. 7 and 8a and Supplementary Table 7). We selected healthy lung tissue from 195 out of 1,346 prospectively recruited treatment-naive patients with lung cancer from the TRACERx cohort (NCT01888601), balancing the cohort for sex, EGFR mutation status and smoking status within the limits of tissue availability (Supplementary Table 7 and Extended Data Figs. 7 and 8a,b). We used ddPCR to detect the presence of five oncogenic EGFR driver mutations (exon 19 deletion, G719S, L858R, L861Q and S768I)^29^ in these tissue samples. We filtered out occurrences where the same mutation was identified in both tumour and non-cancerous tissue using MiSeq-based analysis of corresponding primary tumour tissue (Methods), which were potentially attributable to contamination from the tumour. After this filtering step, 38 out of 195 (19%) patients harboured activating EGFR mutations in healthy lung tissue that were not detectable in tumour tissue (Fig. 4a and Extended Data Fig. 8b). In one patient (identifier CRUK267), both EGFR^L858R^ and EGFR^L861Q^ were detected in healthy lung, but only EGFR^L861Q^ (the less common driver mutation) was found in the tumour. These findings indicate that EGFR-driver mutations can be present in histologically normal lung tissue, even in patients in whom the same mutations were not selected during NSCLC tumorigenesis. To examine whether EGFR mutations exist in healthy lung tissue from people who never develop lung cancer in their lifetime, we profiled 59 healthy lung samples collected at autopsy (median 3 samples per patient, n = 19 patients) from participants in the PEACE study (NCT03004755) who died of other cancers (Supplementary Table 7 and Extended Data Figs. 7 and 8a). An EGFR-driver mutation was detected in the healthy lung of 16% (3 out of 19) patients (Fig. 4a). Despite spatially separated multiregion ddPCR analysis of healthy tissue in 15 out of the 19 patients, EGFR-driver mutations were only detected in 1 region per patient. Based on the frequency of oncogenic EGFR-driver mutations identified in healthy tissue across all patients in the PEACE and TRACERx cohorts (Supplementary Table 7), we used Bayesian inference (Methods) to estimate the presence of an EGFR-driver mutation in lung cells. The calculation showed that 1 in 554,500 lung cells (95% credible interval of 1 in 341,500 to 1 in 865,750 cells) would harbour an oncogenic EGFR mutation. We next used the TRACERx cohort to address whether there was an association of oncogenic EGFR mutations within non-cancerous tissue and exposure to ambient pollution. Anthracosis, determined by the presence of anthracotic pigment (Extended Data Fig. 8c), can act as a surrogate marker of exposure to ambient air pollution^30^. We classified anthracosis within the samples of non-cancerous lung tissue with and without EGFR-activating mutations (Fig. 4b). Although there was no association between the presence of an EGFR-driver mutation in non-cancerous tissue and anthracosis (P = 0.39; Fig. 4b), there was an association between anthracosis and increased variant allele frequencies (VAFs) of EGFR-driver mutations (t-test P = 0.015; Fig. 4c). Although there was a trend towards enrichment of smokers in the anthracosis-positive group (Fisher's exact test, P = 0.065), several reports^30–32^ have shown that cigarette smoking is not a risk factor for anthracosis. In our cohort, the degree of anthracosis observed in never-smokers and smokers did not differ, which is in line with these reports (P = 0.43; Extended Data Fig. 8d). Even though there are multiple environmental contributors to anthracosis^30^, these data suggest that pollutants are not associated with the frequency of activating oncogenic mutations but rather with the expansion of EGFR mutant clones. Smoking status, sex, anthracosis and age of patients in the TRACERx cohort were entered into a multivariable model for the likelihood of anEGFR mutation in healthy tissue. Female sex demonstrated the strongest association (P = 0.06; Extended Data Fig. 8e). We next addressed whether driver mutations existed at other genomic loci in EGFR and in KRAS using an independent ultradeep sequencing platform in a separate group of patients with and without cancer (n = 81). We analysed 48 samples of non-cancerous lung tissue from the PEACE study (lung cancer, n = 9; other cancer, n = 39) and 33 samples of healthy lung tissue derived from the Biomarkers and Dysplastic Respiratory Epithelium (BDRE) study (NCT00900419; Supplementary Table 7 and Extended Data Figs. 7 and 8a). The BDRE cohort consisted of patients with suspicious lung nodules identified through computed tomography scans and who were referred for evaluation by navigational bronchoscopy. For each patient, a brushing sample enriched for bronchial epithelial cells (>89%)^33,34^ from the uninvolved contralateral lung was taken for research purposes and used as the source of healthy tissue. Profiling was carried out using Duplex-seq, which covers a broader range of mutations (EGFR exon 18, 19, 20 and 21, KRAS exon 2 and 3 and other cancer genes). Thus, we only considered mutations featured in the cancer gene census^35^ and further filtered these by evidence of driver mutation status in the literature (Supplementary Table 8). In 24 out of 68 cancer cases for which tissue was available, we also performed Duplex-seq or MiSeq on the corresponding tumour tissue to confirm that the identified mutations were found exclusively in the healthy tissue samples. Based on the Duplex-seq data, 13 out of 81 (16%) samples harboured an EGFR-driver mutation (E709X, G719X, T725M, exon 19 deletion, R765X, R776X, L858R or L861X; Fig. 4d and Extended Data Fig. 9a), whereas 43 out of 81 (53%) samples harboured a KRAS driver mutation (G12X, G13X or Q61X; Fig. 4d and Extended Data Fig. 9b). BRAF inhibitors used to treat BRAF mutant melanomas are known to promote the accelerated growth of clones harbouring RAS mutations^36^. Excluding patients with melanoma from the analysis did not change the percentage of cases harbouring a KRAS mutation (36 out of 68 (53%); Extended Data Fig. 9c), which suggests that this parameter did not confound our analysis. Concordant with KRAS being commonly mutated in ever-smoker LUAD, KRAS mutation frequency and VAFs were significantly higher than EGFR mutation and VAFs in samples from ever-smokers (P = 0.012; Extended Data Fig. 9d). Moreover, VAFs of high-confidence KRAS mutations were consistently higher than those in EGFR in the four ever-smoker cases that harboured oncogenic mutations in both genes (P = 0.015; Extended Data Fig. 9d), which indicates that KRAS mutant clones may be more highly selected for than EGFR mutant clones in healthy lungs of ever-smokers. In summary, 54 out of 295 (18%) of samples of non-cancerous lung tissue harboured an EGFR driver mutation, and 43 out of 81 (53%) samples of non-cancerous lung tissue harboured a KRAS driver mutation. No associations between EGFR or KRAS mutation in non-cancerous tissue and smoking status or cancer diagnosis were observed (Supplementary Table 9). To address whether oncogenic mutations accumulate with the natural ageing process, we examined the driver mutation frequency in all 31 genes (including EGFR and KRAS) present in the Duplex-seq panel. We limited this analysis to 17 never-smoker individuals from the PEACE study to control for any effect of smoking. Consistent with previous work^37,38^, there was a significant correlation between age and mutation count (Fig. 4e).

## Discussion

In this study, we explored the paradigm of tumour promotion driven by the air pollutant PM in the development of lung cancer. We build on previous studies proposing that engine exhaust^39^ and air pollution^40^ induce lung tumours through genotoxicity, induction of oxidative stress and inflammation. We propose that PM can trigger the expansion of pre-existing mutant lung cells through an inflammatory axis that may be amenable to therapy to limit the risk of tumour promotion. Extending previous findings that established associations between air pollution and lung cancer^18^,^41^, including in LCINS^6^, we found an association between the frequency of EGFR mutant lung cancer incidence and increasing PM_2.5_ levels. Temporal analysis suggested that 3 years of PM_2.5_ exposure may be sufficient to increase the risk of developing EGFR-driven lung cancer. A limitation of our epidemiological analysis is its ecological nature: using aggregate data instead of participant-level data. We also acknowledge that variables such as female sex, Asian ancestry and adenocarcinoma histology, which are associated with EGFR mutation status, may confound our conclusions. We balanced our study cohorts with respect to sex and covered geographically and ethnically distinct populations, and when restricting the analysis to LUAD in the English cohort, the positive association remained significant. This study suggests that PM exposure contributes to the observed geographical disparities of EGFR-driven lung cancer, in addition to other established intrinsic (for example, germline genetics^5^) and extrinsic (for example, occupational exposure^3^) factors, and it will be important to understand how these factors interact to increase risk. We observed that PM induces an altered progenitor state in EGFR mutant AT2 cells through the macrophage release of IL-1β, which promotes lung cancer. A caveat of our work is that these mouse models will develop cancers in the absence of PM and probably do not replicate the complex spectrum of mutations found in healthy tissue of a healthy adult. However, they provide controlled environments to provide insight into early tumorigenesis. These experiments demonstrate that a key driver of tumorigenesis is a clinically targetable inflammatory axis that could be applicable to a range of risk factors and malignancies^15^,^42^. It is notable that the antibody canakinumab, which is targeted against IL-1β, a cytokine induced in both mice and humans following PM exposure, has been shown to reduce lung cancer incidence in the cardiovascular prevention trial CANTOS^43^.

A limitation of our DNA profiling strategies of non-cancerous tissue is that we did not purify epithelial cells, specifically AT2 cells, which are the probable initiators of lung tumours. Further work would be required to pinpoint which lineages harbour these mutations. From histological analyses, AT2 and AT1 cells account for on average 22% of distal lung parenchyma cells in autopsy or surgical resection lung samples, mixed with 37% endothelial cells, 37% interstitial cells and 3% macrophages^44^. Our results provide additional evidence that a major trigger of cancer development is not only the inevitable acquisition of driver mutations in healthy epithelium but also intrinsic and extrinsic mechanisms that promote nascent mutant cell expansion and progenitor activity. Assuming little can be done to prevent the acquisition of oncogenic mutations with age, it may be beneficial to address whether additional carcinogens promote cancer through similar inflammatory mechanisms. Broad approaches will be necessary to establish how these carcinogens, as well as potential hormonal, environmental and germline influences, might promote or restrict mutant clone expansions and contribute to tumour promotion. There is an urgent need for carcinogenic assays to identify potential tumour-promoting agents across different tissues and to understand tissue-specific mediators. Such efforts may guide new screening paradigms in high-risk, under-served populations and molecularly targeted cancer prevention approaches to inhibit cancer initiation. In conclusion, our data suggest a mechanistic and causative link between air pollutants and lung cancer, as previously proposed^45^, and substantiate earlier findings on tumour promotion^1^, providing a public health mandate to restrict particulate emissions in urban areas.

## Methods

### ddPCR of tumour and healthy lung tissue samples from the TRACERx and PEACE studies

This project leverages the infrastructure established by the national pan-cancer research autopsy programme (PEACE, NCT03004755) and the prospective, longitudinal cohort study (TRACERx) of NSCLC (NCT01888601)^12^.

To explore whether clinical disparities in lung cancer in never-smokers were reflected in EGFR mutation status in non-cancerous lung tissue, we sought to assemble a cohort comprising participants in the TRACERx study that was as best as possible balanced for sex (male individuals compared to female individuals), smoking status (never-smoker compared with ever smoker) and EGFR mutation status in tumour samples (EGFR mutation versus EGFR wild-type). To uncover whether EGFR mutations were also found in non-cancerous lung tissue from patients who never acquire a lung cancer diagnosis in their lifetimes, we also assembled a cohort of individuals from the PEACE study.

Based on tissue that was available for study, our dataset consisted of 195 tumour and 195 non-cancerous lung tissues from 195 patients from the TRACERx study and 59 non-cancerous lung tissues from 19 participants in the PEACE study (median 3 samples per patient, range of 1–10).

For the TRACERx study, tumour and non-cancerous lung tissue were obtained at surgery. Healthy (non-cancerous) lung tissue was collected distally from the primary tumour tissue (at least approximately 2 cm apart). All tissue was initially snap-frozen and then a portion fixed and made into a formalin-fixed paraffin-embedded (FFPE) block. A haematoxylin and eosin (H&E) section of each block underwent pathology review. DNA was extracted from frozen healthy and tumour tissue proximal to these sections. For the PEACE study, healthy lung tissue was collected at post-mortem tissue from patients who never acquired lung cancer in their lifetimes. Each piece of collected tissue was immediately bisected, and one half was snap-frozen and the other was fixed and made into a FFPE block. The H&E section of each block underwent pathology review. DNA was then extracted from an adjacent frozen healthy tissue sample.

All aforementioned H&E slides from tissues underwent central pathology review. In particular, to exclude the possibility of contamination with tumour cells, thoracic pathologists confirmed that all healthy lung tissue samples did not contain any indication of tumour tissue or morphologically defined, pre-invasive disease. Thoracic pathologists also identified anthracotic pigment and reflected this in a binary score for its presence. For anthracosis-positive cases, the proportion of the tissue covered by anthracotic pigment was quantified.

### EGFR mutation profiling of non-cancerous tissue samples by ddPCR

DNA was extracted from healthy lung tissue samples as previously described^12^. The DNA concentration was measured using Qubit, and up to 3,000 ng of DNA was fragmented to approximately 1,500 bp using a Covaris E220 evolution focused-ultrasonicator following the manufacturer's standard protocol. SAGAsafe assays^46^ for five EGFR target variant alleles (L858R, exon 19 deletion, S768I, L861Q and G719S) were used (SAGA Diagnostics). SAGAsafe is a digital PCR-based ultrasensitive mutation detection technology that utilizes an alternative chemistry alongside a modified thermocycling program, such that the true positive variant allele signal is enriched during a linear phase, and signals for both the variant and the wild-type alleles are amplified during the exponential phase. The method effectively suppresses the false-positive variant allele signal arising from polymerase base misincorporation errors and DNA damage, making reliable detection of rare-event mutations possible to exceedingly low limits of detection. The assays were performed on a Bio-Rad QX200 Droplet Digital PCR system. At least three positive droplets were required to call a sample positive. Using control experiments containing 265,000–381,000 copies of wild-type genome equivalents per test, the achievable limit of detection for the five EGFR SAGAsafe assays was determined to be at least 0.004% VAF. For each patient sample, 500 ng of fragmented DNA (corresponding to about 150,000 copies of genome equivalents) was analysed per assay across 4 reaction wells, with positive and negative control samples included for every run.

The copy number concentration of the variant and the wild-type alleles was calculated as follows:

$$C_{V_{i}}=\frac{-\ln(1-\frac{P}{T})}{V_{d}}\times \frac{V_{r}}{V_{i}}$$

where C_Vi_ is the copy number concentration of the target (variant or wild-type allele) in the input DNA sample, P is the number of positive droplets for the target, T is the number of total droplets analysed, V_d_ is the volume a droplet (0.85 x10^−3^ μl), V_r_ is the total volume of a ddPCR reaction (20 μl), and V_i_ is the input volume per ddPCR reaction of the input DNA sample.

The VAF was calculated as follows:

$$VAF=\frac{C_{V_{i}}^{\text{Variant}\;}}{C_{V_{i}}^{\text{Variant}\;}+C_{V_{i}}^{Wild-type\;}}\times 100\%$$

To estimate the EGFR mutation rate, we considered all five oncogenic EGFR mutations detected by ddPCR in all TRACERx and PEACE samples analysed (253 samples in total). Using the approximate Bayesian computation model, we simulated ddPCR results of oncogenic EGFR mutations and inferred a mutation rate of 4.07 x10^−7^ per mutation (confidence interval: 1.61 x 10^−7^ to 6.08 x 10^−7^). Considering this mutation rate, we estimated that the frequency of identifying 1 EGFR mutation (of any of the 5 mutation types) would be 1 in 2,035,000 (95% confidence interval: 1 in 805,000 to 1 in 3,040,000). When we took the average of the two limits of the confidence interval, we obtained an estimate of an EGFR mutation being present in 1 in 554,500 cells (or around 1:600,000 cells).

### EGFR mutation profiling in corresponding tumour tissue by MiSeq

To exclude the presence of clonal or subclonal spatially distinct EGFR mutations that may be present in the corresponding matched lung tumour, we performed multiregion deep next-generation sequencing of NSCLC samples from the same patients (>3,000x coverage) of 19 driver genes (including EGFR) using the MiSeq platform. We sequenced 751 tumour regions from the 195 tumours (median of 3 regions per tumour) with an achievable limit of detection in each tumour region of 0.966% based on a median sequencing depth per region of 3,490x and a MiSeq error rate of 0.473%^47^.

For each tumour region and matched germline, capture of a custom panel of genes (including the EGFR locus) was performed on 125 ng DNA isolated from genomic libraries. The TruSeq Custom Amplicon Library Preparation method was used. Following cluster generation, samples were 100 bp paired-end multiplex sequenced on an Illumina MiSeq platform at the GCLP Laboratory at University College London, as previously described^12^. The generated data were aligned to the reference human genome (hg19). Mutations were called as previously described^12^.

### Duplex-seq of samples from the PEACE and BDRE studies

#### Non-cancerous lung tissue samples

Samples from the PEACE cohort were collected as described above. For Duplex-seq, we obtained additional non-cancerous lung tissue from 48 participants of the PEACE study. Here patients with lung cancer or with another cancer type were profiled (lung cancer, n = 9; other cancer, n = 39).

Participants in the BDRE study (NCT00900419) consisted of individuals recommended for a computed tomography (CT) scan based on age, smoking history or other indications. If a suspicious nodule was detected by CT scan, a navigational bronchoscopy was indicated. The nodule site was sampled for accurate diagnosis. For each patient, a brushing from a remote site in a contralateral lobe was also taken for research as a representative sample of non-cancerous tissue and subsequently profiled for mutations using Duplex-seq. The absence of nodules or masses detected by chest CT scans was indicative of the non-tumour nature of these contralateral samples. Each procedure was performed under fluoroscopic guidance, with the brush advanced from the sheath only after documentation that the working channel was in the peripheral airways.

#### EGFR and KRAS mutation profiling by Duplex-seq

Genomic DNA was extracted from brushing samples using a Qiagen DNeasy Blood and Tissue kit according to the manufacturer's instructions. Duplex libraries were prepared using a commercially available kit from Twin-Strand Biosciences (CKD-00042 panel 000323), starting with 250 ng of input DNA. Custom probes were designed for targeted capture of EGFR exons 18, 19, 20 and 21, and KRAS exons 2 and 3, along with 29 other cancer genes.

By independently capturing and sequencing the two strands of DNA for selected genomic regions, combined with the use of a common unique molecular identifier for both strands, Duplex-seq enables the detection of rare mutations^46^ with a sensitivity of less than 1 in 10^7^. After shearing and capturing of gDNA spanning the panel, primers were ligated so that the two strands of DNA for each segment were uniquely labelled and matched with its opposing strand. These strands were then amplified, and libraries were sequenced on a NovaSeq 6000 sequencing system (Illumina), and sequencing data were processed using a DNAnexus platform. Samples had an average number of 150,000,000 raw reads, producing a mean on-target duplex depth of 4,500. Duplex-seq reads were processed using a previously published pipeline^48^, similar to a bioinformatics pipeline provided by TwinStrand BioSciences. Using this, we were able to identify mutations that were present in both the involved and contralateral lung samples.

## Epidemiological studies

### UK Biobank dataset

The UK Biobank (UKBB) study comprises more than 500,000 participants, aged between 37 and 73 years, who were recruited between 2006 and 2010. Participants provide detailed information regarding a comprehensive set of lifestyle factors, in addition to physical measurements and biological samples. PM air pollution levels (in 2010) were estimated for addresses within 400 km of the Greater London monitoring area using a land-use regression model developed as part of the ESCAPE study^49^.

Lung cancer cases were those with International Classification of Diseases (ICD; tenth revision) codes C33 or C34. Associations between PM_2.5_ levels and lung cancer incidence in the UKBB data have already been calculated and previously reported^18^.

We accessed the UKBB data under project number 82693. Ethical approval of the UKBB study was given by the North West Multicentre Research Ethics Committee, the National Information Governance Board for Health and Social Care, and the Community Health Index Advisory Group.

To impute missing data, we first excluded all participants who had any cancer diagnosis pre-recruitment, or a cancer diagnosis date entry but no corresponding cancer annotation, alongside those with missing particulate matter or genetic principal components data. Multiple imputation with chained equations^50^ was used to impute missing smoking status (categorized into never, previous and current; <1% missing), passive smoking (weekly hours of home tobacco exposure; 10.0% missing), pack-years of smoking (15.4% missing), body–mass index (BMI) (<1% missing), household income (dichotomized by ≥GBP£31,000 annually; 14.6% missing) and educational attainment (split by degree or professional qualification status; 1.31% missing) values. In addition to these variables, imputation models used the following variables to predict values for missing data: PM_2.5_, age at baseline, sex, BMI and the first 15 genetic PCs (to account for ethnicity). These were used alongside cancer outcome and duration of follow-up. We used predictive mean matching, logistic regression and random forest for continuous, binary and categorical variables, respectively, performing a maximum of 180 iterations for the generation of each imputed dataset. This produced 15 complete versions of the original dataset in which the missing values were imputed. This dataset comprised 407,509 individuals and represented 28 cancer types. Each imputed dataset was independently used in the same analysis protocol.

Participants were followed up from recruitment until either date of each cancer diagnosis (obtained through linkage to national cancer registries) or censoring, which was defined as time of death, lost to follow-up or the end of 2018, whichever was earlier. We created a multivariate Cox regression model for each imputed dataset and primary cancer type with ≥100 cases (excluding non-melanoma skin cancer, and cancers restricted to a single sex), and pooled results across these models, which were consistent for each cancer type, into a single set using Rubin's rules^50^. Confidence intervals were calculated using e^estimate_pooled_+-(1.96xstandarderrorpooled)^. These models included the same covariates as in the imputation model. For laryngeal alongside lip and oropharyngeal cancers, we further corrected for alcohol consumption, excluding those participants with missing alcohol data owing to the high missingness of these variables (30.7%). Schoenfeld residuals were examined to assess the proportional hazards assumption, with non-proportionality confirmed using Kaplan–Meier curves for binary and categorical variables. Potential departures from the proportional hazards assumption were noted for anal (smoking status), bladder (genetic PC 12), kidney (age and smoking status) and melanoma (genetic PC 9 and sex). We note high median (across all 15 imputations) variance inflation factor values (≥5) for the following covariates: genetic PC 1 (other and unspecified biliary tract parts); PC 2 (acute myeloid leukaemia, follicular nodular non-Hodgkin lymphoma, larynx, mesothelioma, other and unspecified biliary tract parts, peripheral and cutaneous T lymphomas, retroperitoneum and peritoneum); and PC 3 (acute myeloid leukaemia, follicular nodular non-Hodgkin lymphoma, larynx, mesothelioma, other and unspecified biliary tract parts, peripheral and cutaneous T lymphomas). Finally, we report FDR-corrected P values for the association between PM_2.5_ levels and cancer incidence to account for multiple testing.

Our methods differed from those of Huang et al.^18^ in the following ways: (1) we increased the number of imputations from 5 to 15 and iterations from 90 to 180; (2) we augmented our multivariate analysis to better account for the effect of smoking by categorizing participants into never, previous and current smokers, and included passive smoking; (3) we included the first 15 genetic PCs in our multivariable analysis of PM_2.5_ and cancer incidence. An interaction test between PM_2.5_ and smoking was then performed for lung cancer, considering only participants with complete covariate data in the multivariable Cox regression. For the LUAD-specific analysis, we considered only participants with cancer registry histology entries that map to LUAD (Supplementary Table 4). Imputations and all downstream modelling was performed independently for this analysis. To take into account migration, as the PM_2.5_ data are available for each participant's address, we assumed that participant PM_2.5_ exposure levels remained constant throughout the study period. To account for exposure misclassification, we additionally performed a separate analysis that included only participants who had lived at their current address for at least 3 years before baseline. All imputations and downstream analysis was performed independently for this subgroup.

Radon exposure data from the British Geological Survey (BGS) was merged with the UKBB dataset based on home location coordinates. As the data from BGS had greater spatial resolution, values were aggregated by the mode radon potential class (breaking ties through taking the higher class value) across all BGS coordinate values that map to each rounded coordinate in the UKBB. Imputations and downstream analyses were performed as described above, using modal radon exposure instead of PM_2.5_.

### Comparison of the UKBB population with the general UK population

Estimated HRs from UKBB analyses are higher than in some population-based epidemiological surveys^41^, which may reflect over-representation of less wealthy, never-smoker individuals in the UKBB. We have provided a table (Supplementary Table 4) comparing some characteristics between the UKBB population we studied and UK population estimates for reference. Compared with the general population, UKBB participants consisted of fewer current smokers, were more highly educated, had lower household income, were more likely to be female individuals, older, white and to live in areas with lower PM^2.5^ levels.

### Within-country datasets England dataset (NDRS)

Air pollution, lung cancer incidence and EGFR mutation status could be estimated for 20 Cancer Alliance regions in England. This was the geographical level at which all three factors could be quantified. Annual PM_2.5_ air pollution data (μg m^−3^) from 2006 to 2017 was obtained at the grid code level (1x1 km) from DEFRA^51^. Radon potential (defined as the estimated percentage of homes in an area above the radon action level) in 2011 was obtained from the British Geological Survey at the grid code level^52^. Postal code coordinates were sourced from the Office of National Statistics 2018 Postal Code Directory^53^. To link every postal code to a grid code with pollution data, the coordinates of every postal code centroid was mapped to those of the nearest grid code centroid using the RANN package in R. The postal codes with pollution data were binned into 1 of 20 Cancer Alliance regions. Then PM_2_.5 concentration estimates were aggregated to the Cancer Alliance region level and then averaged over the period 2008–2017 for 2018 diagnoses, 2007–2016 for 2017 diagnoses and 2006–2018 for 2016 diagnoses—these were selected because they represented the 10 years before a lung cancer diagnosis. The air pollution levels in each Cancer Alliance region were broadly stable (within 5 μg m^−3^) in this time period. Incidence data on 118,019 (2016, 39,229; 2017, 39,500; 2018, 39,290) lung cancers (ICD codes C33 to C34) diagnosed in England between 1 January 2016 and 31 December 2018 were extracted from the National Cancer Registration Dataset (AV2018 in CASREF01 (end of year snapshot)), held by the National Disease Registration and Analysis Service at England's NDRS. Lung cancer incidence for each Cancer Alliance region was calculated based on these cases. This represented a predominantly white cohort: white, 92.06%; Asian, 1.48%; Chinese, 0.23%; Black, 1.05%; mixed: 0.28%; other: 0.94%; unknown: 3.96%. The age-standardized lung cancer incidence (using population counts obtained from the Office of National Statistics 2019 (2018 mid-year estimates)) was obtained according to each 5-year age group and sex. Incidences were then combined across age and sex to produce a single value for each Cancer Alliance region as follows: lung cancer incidence = (sum(wi × xi/di)/sum(wi)) × 100,000. Where wi is the European population standard, di is the population count and xi the case count. Standardized rates were standardized according to the 2013 European Standard Population. Confidence intervals for age-standardized rate point estimates were calculated using the Dobson method. For lung cancer diagnoses listed above, EGFR mutation statuses were extracted from the National Cancer Registration Dataset (AT_GENE_ ENGLAND table in the CAS2210 monthly snapshot), which includes data on somatic tests undertaken from 1 January 2016 to 31 December 2019. Only cases with ‘Overall: TS’ as ‘a:abnormal’ and ‘b:normal’ for EGFR were used in the calculation for the EGFR mutation rate (n = 25,567). The EGFR mutation rate was calculated for each Cancer Alliance region as follows: EGFR mutation rate = [number of a:abnormal]/[(number of a:abnormal) + (number of b:normal)]. The NDRS data included in this study were collected and analysed under the National Disease Registries Directions 2021, made in accordance with sections 254(1) and 254(6) of the 2012 Health and Social Care Act. Further ethical approval for this study was not required per the definition of research according to the UK Policy Framework for Health and Social Care Research.

### South Korea dataset (Samsung Medical Center)

Air pollution, lung cancer incidence and EGFR mutation status could be estimated for 16 geographical regions in South Korea. This was the geographical level at which all three factors could be quantified. PM_2.5_ air pollution data were obtained from Air Korea^54^ for the years 2015–2017 for 16 standard geographical regions across Korea. Within each of the geographical regions, we averaged PM_2.5_ levels across the 2-year period before the year of lung cancer diagnosis. PM_2.5_ levels between 2015 and 2017 were broadly stable. We were only able to include PM_2.5_ data for a 2-year period for 2017 and 2018 diagnoses, as air pollution data per region in Korea was only available starting from 2015. Lung cancer incidence data were obtained from the Korean National Cancer Center^55^ for the years 2017 to 2018 for 16 geographical regions across Korea. Sex and smoking data were not available. Lung cancer incidence was obtained separately for each year and considered independently in Pearson correlations that are described below. Lung cancer EGFR mutation status was obtained from Samsung Medical Center lung cancer diagnoses for the years 2017 to 2018 for 16 geographical regions across Korea (n = 2,563). The EGFR mutation rate was calculated as described above. The study was conducted under an institutional review boardapproved protocol (number 2021-06-043) at the Samsung Medical Center.

### Taiwan dataset (Chang Gung Medical Foundation)

Air pollution, lung cancer incidence and EGFR mutation status could be estimated for 12 standard geographical regions in Taiwan. This was the geographical level at which all three factors could be quantified. Annual PM_2.5_ air pollution data were obtained for 12 standard geographical regions in Taiwan from the Environmental Protection Administration Executive Yuan R.O.C. (Taiwan)^56^. PM_2.5_ (μg m^−3^) concentration estimates were available for each county in Taiwan from 2006 to 2017. We averaged PM_2.5_ levels across the period (up to 10 years before a 2-year washout period) before the year of lung cancer diagnosis. For example, for a diagnosis in 2017, 2006–2015 aggregated air pollution levels were used for analysis, whereas for a diagnosis in 2011, 2006–2009 aggregated air pollution levels were used for analysis. A 2-year washout period was necessary to account for substantial decreases in air pollution levels after 2013. Institutional lung cancer incidence and EGFR mutation rates for each of 12 different counties in Taiwan were obtained from the Chang Gung Research Database for the years 2011–2017 (n = 4,599). Lung cancer incidence was obtained separately for each year and considered independently in Pearson correlations that are described below.

Institutional lung cancer incidence was estimated based on recorded lung cancer diagnoses in all of Chang Gung Medical Foundation hospitals, and the age-standardized rates per 100,000 were calculated using the world (World Health Organization 2000) standard population of lung cancer incidence. EGFR mutation testing data were available for all of these cases. However, only nine counties had at least ten cases with EGFR mutation tested per year and constituted >5% of the total population; these were the counties that were retained for analysis. The EGFR mutation rate was calculated as outlined above. The data from the Taiwan cohort was from the Chang Gung Research Database, which is approved by the institutional review board of Chang Gung Medical Foundation (202101202B0).

### Relationship between EGFR mutant lung cancer incidence and PM_2.5_

Analyses were performed separately for each of the three cohorts: England, South Korea and Taiwan. For each geographical region (for example, each country or the 20 Cancer Alliance regions in England), EGFR-driven lung cancer incidence was calculated by multiplying the total lung cancer incidence by the EGFR mutation rate (as reported as a proportion out of 1) as follows: EGFR mutation lung cancer incidence = lung cancer incidence x EGFR mutation rate. EGFR mutant lung cancer incidence values were compared with mean PM_2.5_ values across geographical regions using Pearson correlation tests, weighted Pearson correlation tests (to account for number of tested cases in each geographical region) and robust linear regression (to account for outliers).

### Sensitivity analysis for the England and Korea datasets

In the England dataset, there were two Cancer Alliance regions (South East London and Thames Valley) with sparse data owing to data unavailability (<5% of lung tumours diagnosed in 2016–2018 have a definitive test result recorded for EGFR). To exclude the possibility of this confounding our analysis, we performed a sensitivity analysis, whereby we excluded data from these two regions. Of note, the correlation between PM_2.5_ and EGFR-driven lung cancer incidence was still significant (r = 0.55, P = 0.019) after these exclusions. Similarly, in the South Korea dataset, Jeju-do (2017) was excluded owing to poor data availability. The correlation between PM_2.5_ and EGFR-driven lung cancer incidence was still significant (r = 0.38; P = 0.033) after this exclusion. However, for the sake of completion, we report the full datasets (including these two regions in England regions and one region in South Korea region) in the main text.

### Canada dataset (BC Cancer Research Centre, Vancouver BC, Canada)

This dataset comprises 228 lung cancer cases from female patients and has been previously reported^6^. These patients were seen at the Thoracic Surgery Department of the Vancouver General Hospital or the BC Cancer Vancouver Cancer Center between 15 November 2017 and 31 May 2019, and were prospectively invited to take part in the study. Detailed residential histories from birth to cancer diagnosis for residences within Canada and previous residences outside of Canada (for foreign-born immigrants) were recorded. Street and city address or postal codes enabled accurate linking of residential locations to satellite-derived PM_2.5_ exposure data that were available from 1996 onwards. A personal PM_2.5_ cumulative exposure value was individually calculated using a detailed residential history from birth to current address, and input into Geographical Information System mapping. By applying high-resolution (10 x 10 km) concentration estimates of PM_2.5_ from satellite observations, chemical transport models and ground measurements to each individual's residential history, a cumulative exposure value was estimated by taking into account the intensity and duration of exposure and summing over all residences. EGFR mutation status for each patient was obtained from each patients’ hospital record. This study was approved by the UBC_BC Cancer Research Ethics Board.

### Defining pollution exposure groups

Low, intermediate and high air pollution groups were defined by considering quintiles of the distribution of PM_2.5_ exposure levels across the entire dataset (3 years of cumulative pollution data and 20 years of cumulative pollution data). The following thresholds were applied: bottom quintile, 6.77 μg m^−3^; top quintile, 7.27 μg m^−3^; PM_2.5_ low, PM_2.5_< bottom quintile; PM_2.5_ intermediate, PM_2.5_> bottom quintile and PM_2.5_<top quintile; PM_2.5_ high, PM_2.5_> top quintile.

### Comparing EGFR mutant frequencies

EGFR mutation frequencies were compared between high and low pollution exposure groups using chi-squared tests. Two comparisons were performed: high versus low pollution (based on 3 year data) and high versus low pollution (based on 20 year data).

## Preclinical studies

### Animal procedures

Animals were housed in ventilated cages with unlimited access to food and water. All animal regulated procedures were approved by The Francis Crick Institute BRF Strategic Oversight Committee, incorporating the Animal Welfare and Ethical Review Body, conforming with UK Home Office guidelines and regulations under the Animals (Scientific Procedures) Act 1986 including Amendment Regulations 2012. Both male and female mice aged 6–15 weeks were used. EGFR^L858R^ (Tg(tet-O-EGFR*L858R)56Hev) mice were obtained from the National Cancer Institute Mouse Repository. Rosa26tTA and Rosa26-LSL-tdTomato mice were obtained from the Jackson laboratory. Mice were backcrossed onto a C57Bl6/J background and further crossed to generate Rosa26^LSL-tTa/LSL-tdTomato^;TetO-EGFR^L858R^ mice. CCSP-rtTa;TetO-EGFR^L858R^ and Rosa26^LSL-tTa/LSL-tdTomato^;Kras^LSL^-^G12D^ mice have been previously described^57^,^58^. After weaning, the mice were genotyped (Transnetyx) and placed in groups of 1–5 mice in individually ventilated cages, with a 12-h daylight cycle. Cre-mediated recombination was initiated by adenoviral CMV-Cre (Viral Vector Core) delivered by intratracheal intubation (2.5 x 10^7^ virus particles per 50 pl), by Ad5-SPC-Cre (Viral Vector Core, donated by A. Berns) delivered by intratracheal instillation (2.5 x 10^8^ virus particles per 50 μl)^21^ or by using chow containing doxycycline obtained from Harlan-Tekland. For antibody treatment, mice were given 200 μg of anti-mouse/rat IL-1β (B122, InVivoMAb, BE0246) or rat IgG control (InVivoMAb, BE0091) by intraperitoneal injection on the same day as PM exposure.

For exposure to fine PM or PBS, SRM2786 from the National Institute of Standards and Technology (obtained from Sigma Aldrich) was resuspended in sterile PBS using sonication, and the particle size distribution was confirmed using a dynamic light scattering analyser (Zetasizer, mean particle diameter 2.8 μm). SRM2786 has certified mass fraction values of both organic and inorganic constituents from multiple analytical techniques and represents fine PM from a modern urban environment^59^. Mice were briefly anaesthetized using 5% isoflurane followed by intratracheal administration of 50 μg or 5 μg in a volume of 50 μl (ref. 60). Mice were intratracheally administered with PM or PBS three times per week for 3 weeks with at least 48 h between each administration. FACS analysis and cell sorting. For flow cytometry analysis of immune cells, mouse lungs were minced into small pieces, incubated with collagenase (1 mg ml^−1^; ThermoFisher) and DNase I (50 U ml^−1^; Life Technologies) for 45 min at 37 ÅãC and filtered through 100 .m strainers (Falcon). Red blood cells were lysed for 5 min using ACK buffer (Life Technologies). Cells were stained with fixable viability dye eFluor780 (BD Horizon) for 30 min and blocked with CD16/32 antibody (BioLegend) for 10 min. Cells were then stained with antibodies for 30 min (Supplementary Table 10). Intracellular staining was performed using a Fixation/Permeabilization kit (eBioscience) according to the manufacturer's instructions. Samples were resuspended in FACS buffer (2% FCS in PBS) and analysed using a BD Symphony flow cytometer. Data were analysed using FlowJo (Tree Star).

**Extended Data Figure 6:**
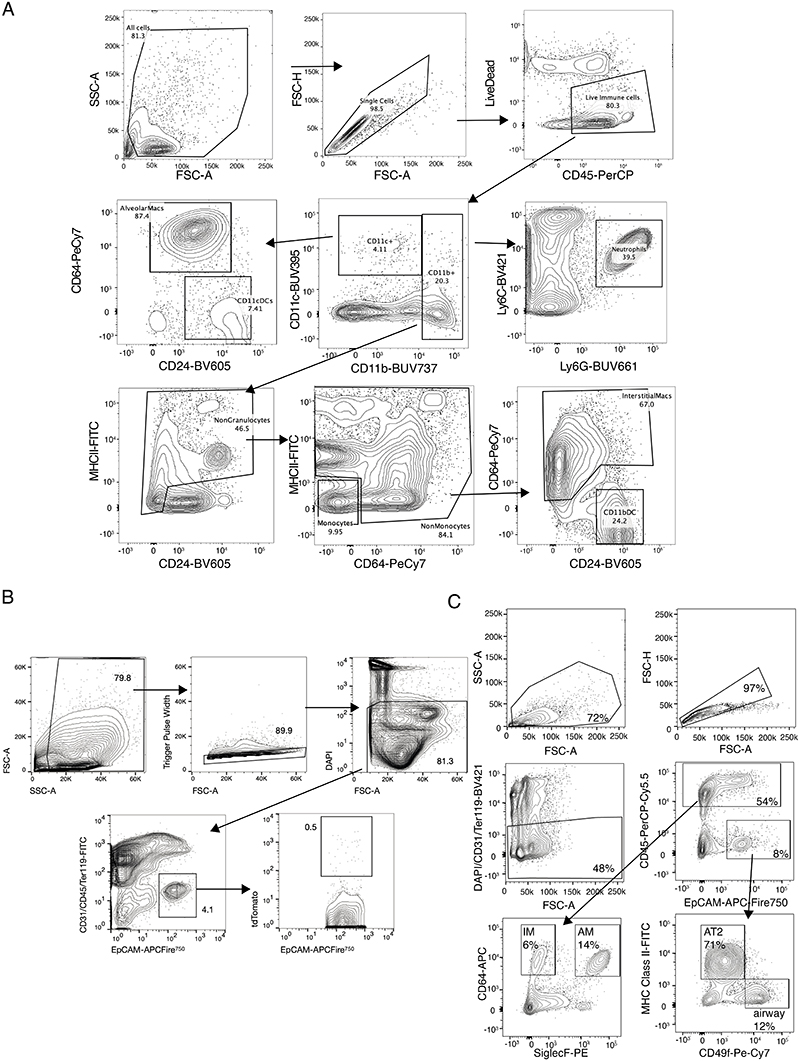
A, B) Example of flow gating strategy to determine frequency of lung (A) alveolar macrophages, interstitial macrophages, neutrophils, dendritic cells and (B) epithelial cells both tdTomato positive and negative. All samples were first gated to exclude debris and doublets, followed by live cell discrimination. C) Representative picture from a tdTomato mouse treated with control PBS for 3 weeks using sort strategy to enrich for for AT2 cells defined in Major et al., 2020 and both alveolar and interstitial macrophages defined in Choi et al., 2020

For flow cytometry sorting of AT2 cells^61^, epithelial cells and immune cells, minced lung tissue was digested with Liberase TM and TH (Roche Diagnostics) and DNase I (Merck Sigma-Aldrich) in HBSS for 30 min at 37 °C in a shaker at 180 r.p.m. Samples were passed through a 100 μm filter, centrifuged (300g, 5 min, 4 °C) and red blood cells were lysed as described above. Extracellular antibody staining was then performed followed by incubation in DAPI (Sigma Aldrich) to label dead cells. Gating strategies for sorting and analysis are outlined in Extended Data Fig. 6. Cell sorting was performed on Influx, Aria Fusion or Aria III instruments (BD).

### Immunohistochemistry

Mouse lungs were fixed overnight in 10% formalin and embedded in paraffin blocks. Then 4 μm tissue sections were cut, deparaffinized and rehydrated using standard methods. Antigen retrieval was performed using pH 6.0 citrate buffer and incubated with antibodies (Supplementary Table 10). Primary antibodies were detected either using biotinylated secondary antibodies, followed by HRP or DAB, or with subsequent OPAL fluorescence secondary antibodies (Akoya). A commercial kit was used to detect IL1B RNA transcripts by RNAscope (ACD Biotechne) following the manufacturer's instructions. Staining for CD68 protein was subsequently performed and detected using OPAL fluorescence following the manufacturer's protocols (Akoya). Probes visualized through fluorescence were used to detect IL-1β RNA and CD68 protein simultaneously. Slides were imaged using a Leica Zeiss AxioScan.Z1 slide scanner. Tumour grading and lesion analysis was carried out by two board certified veterinary pathologists. EGFR mutant cell foci were quantified from cell coordinate data by clustering cell positions by density using the DBSCAN algorithm, implemented in Python with the scikit-learn library^62^. We chose an EPS value of 35 for DBSCAN clustering as this produced spatial clusters with excellent concordance to visual inspection of foci in the original histological images. To assess the fraction of clusters that had expanded, we reasoned that wild-type cells may divide only once between 3 and 10 weeks, which is based on the low proliferation rate of alveolar epithelial cells^63^. As there was an average cluster size of 2 EGFR mutant cells at 3 weeks, we defined clusters of >5 cells at 10 weeks as ‘expanded clusters’ that expanded above expected. Segmentation and analysis of immunohistochemistry and immunofluorescence images was carried out using QuPath^64^.

### Whole-genome sequencing

ET lung tumours from PBS-treated mice (n = 5) and PM-exposed mice (n = 5) were collected at ethical end points. Individual lung tumours were dissected from lung lobes and snap frozen. Germline DNA was extracted from t ail tissue. DNA was isolated and prepared for whole-genome sequencing (WGS), which was followed by sequencing on a NovaSeq instrument (Illumina) to achieve target coverage of 100x for PBS-treated and PM-exposed samples and 30x for germline samples. Sequences from all 20 samples were processed using the Nextflow (v.21.10.3) Sarek pipeline (nf-core/sarek v.3.0). In brief, sequences were aligned with BWA (v.0.7.17) to mm10, and mutations were called using Mutect2 (gatk4: 4.1.8.1). Only mutations labelled as ‘PASS’ by Mutect2 that were uniquely present in each tumour were considered for analysis. Mutational signatures were called using the DeconstructSigs R package^65^, restricting our analysis to the following common SBS signatures: SBS1, SBS4, SBS5, SBS2, SBS13, SBS40, SBS92, SBS17a, SBS17b and SBS18.

### RNA-seq

CD45^−^CD31^−^TER119^−^EpCAM^+^ lung cells from PBS-treated and PM-exposed mice were sorted by flow cytometry. Total RNA was isolated using a miRNeasy Micro kit (Qiagen) according to the manufacturer's instructions. Library generation was performed using KAPA RNA HyperPrep with RiboErase (Roche), followed by sequencing on a HiSeq (Ilumina) instrument to achieve an average of 25 million reads per sample. The RNA-seq pipeline of nf-core framework (v.3.3) was launched with Nextflow (v.21.04.0) to analyse RNA-seq data^66^. Raw reads in fastq files were mapped to GRCm^38^ with associated ensemble transcript definitions using STAR (v.2.7.6a)^67^. BAM files were sorted with a chromosome coordinate using samtools (v.1.12). RSEM (v.1.3.1) was used to calculate estimated read counts per gene and to quantify a measure of TPM^68^. Differential expression analysis was performed using the R platform (v.4.0.3) package DESeq2 (ref. 69), filtering with the absolute value of log(fold change) > 1 and FDR < 0.05. Significantly differentially expressed genes were determined using a generalized linear model within DESeq2 and a Wald test. Gene expression levels between treatment groups was further analysed for their pathway enrichments using gene set enrichment analysis^70^. Normalization (using z-scores) of TPM scores across the dataset was performed before plotting heatmaps of gene expression. The AT2 activated score was derived using a previously described method^71^. In brief, bulk RNA-seq data from mouse models, with or without an EGFR mutation and in the presence or absence of PM exposure, were compared according to the degree to which they were similar to a signature of activated AT2 transitional progenitor cells (‘AT2 activated’) derived from previously published single-cell RNA-seq data^23^. This signature was estimated using a pseudoR2 value calculated using a previously described approach^71^. This approach was adapted to a mouse dataset using gene weights from mouse-to-human orthologous genes. The pseudoR2 value was used as a continuous input in a test between the different conditions. Comparison of RNA-seq data from mice to never-smokers in the COPA study. RNA-seq was applied to 18 samples of bronchial brushings from nine never-smokers from the COPA study after exposure to filtered air and diesel exhaust. Salmon^72^ was used to estimate transcript-level abundance from RNA-seq read data. Differential expression analysis was performed using DESeq2 (ref. 69). The log twofold difference in gene expression was calculated between samples collected 24 h after exposure to diesel exhaust and filtered air (control) on separate occasions but from the same participants. P values were adjusted using the Benjamini–Hochberg method. The log twofold change of significantly differentially expressed genes between the tdTomato control and td-Tomato PM-treated mice were compared to the log twofold change expression of the genes from COPA participants. All participants in the COPA study provided informed consent. The consent forms and study protocol were approved by the University of British Columbia Clinical Research Ethics Board (number H12-03025), Vancouver Coastal Health Ethics Board (number V12-03025) and Health Canada's Research Ethics Board (number 2012–0040). The limitation of this analysis is that the mouse and human RNA-seq datasets fundamentally differ in the following ways. (1) Mouse data were acquired from total lung EpCAM^+^ cells, containing both airway and alveolar tissue, whereas the human data were obtained from bronchial brushings only; therefore, different cell types are represented in the data. (2) The pollution exposure between species differed. Human participants were exposed to diesel exhaust for 2 h compared to 3 weeks of PM exposure for mice. Furthermore, the mice were kept in controlled environments, whereas a 4-week washout period between exposure to filtered air and diesel exhaust in human participants was required, where day-to-day PM exposures and lifestyle differences could not be controlled. (3) Fold changes from the human data were obtained by pairwise comparisons from each individual. By contrast, because we did not have pairwise matched data from each mouse, the fold changes from the mouse data were derived based on aggregated (mean) values across each condition (that is, air pollution versus control). (4) The RNA-seq was performed at two different sequencing centres and target depths were different. The human data were sequenced with a target depth of 30 million reads per sample, whereas the mouse data were sequenced with a target depth of 25 million reads per sample.

### Organoid-forming assays

Lung organoid co-culture assays have been previously described^22^. In brief, tdTomato^+^ lung epithelial cells (tdTomato^+^EpCAM^+^CD45^−^CD31^−^TER119^−^) and tdTomato^−^ lung epithelial cells (tdTomato^-^EpCAM^+^CD45^−^CD31^−^TER119^−^) were isolated by FACS from PBS-treated or PM-exposed ET mice after 3 weeks of treatment and were resuspended in 3D organoid medium consisting of DMEM/F12 with 10% FBS, 100 U ml^−1^ penicillin–streptomycin, insulin, transferrin, selenium, l-glutamine (all from Gibco) and 1 mM HEPES (in-house). About 5,000–10,000 cells were mixed with a mouse lung fibroblast cell line (MLg2908, American Type Culture Collection, 1:5 ratio) and resuspended in growth-factor-reduced Matrigel (Corning) at a ratio of 1:1. Next 100 μl of this mixture was pipetted into a 24-well Transwell insert with a 0.4 μm pore (Corning). After incubating for 30 min at 37 μC, 500 μl of organoid medium was added to the lower chamber and the medium changed every other day. Bright-field and fluorescence images were acquired after 14 days using an EVOS microscope (Thermo Fisher Scientific) and quantified using Fiji (v.2.0.0-rc-69/1.52r; ImageJ). For ex vivo IL-1β treatment of lung AT2 cells, single-cell suspensions from ET mice lungs (without in vivo Cre induction) were subject to AT2 cell purification as previously described (MHC Class II^+^CD49f^low^ EpCAM^+^CD45^−^CD31^−^TER119^−^)^61^. Purified AT2 cells were incubated in vitro with 6 x 10^7^ p.f.u. ml^−1^ of Ad5-CMV-Cre in 100 pl per 100,000 cells in 3D organoid medium for 1 h at 37 μC as previously detailed^27^. Cells were washed three times in PBS before plating as described above, and 20 ng ml^−1^ IL-1β was added to the organoid medium in the lower chamber and changed every other day. TdTomato^+^ organoids were quantified in Fiji. For whole-mount staining of organoids, organoids were prepared according to previous published methods^73^ and stained with anti-proSPC (Abcam, clone EPR19839) and anti-KRT8 (DSHB Iowa, clone TROMA-1). 3D confocal images were acquired using an Olympus FV3000 microscope and analysed using Fiji. For assessment of AT2 organoid formation after PM exposure, AT2 cells were isolated from PBS-treated or PM-treated control mice and ET mice after 3 weeks, without in vivo Cre induction. Following Cre infection, 10,000 cells were plated in the organoid assay as described above. For co-culture of AT2 cells and macrophages, non-induced ET mice were exposed to either PBS or PM, followed by collection at 3 weeks, and AT2 cells, interstitial cells and alveolar macrophages were isolated as previously detailed^22^ (sorting strategies are defined in Extended Data Fig. 6c). AT2 cells from PBS-treated ET mice only were infected with Cre ex vivo as described above, before 10,000 AT2 cells were either plated with fibroblasts only or with a 1:6 ratio of PBS-treated or PM-treated macrophages as described above, modified from a previously published method^22^. tdTomato^+^ organoids were quantified in all conditions.

## Statistics and reproducibility

Preclinical statistical analyses were performed using Prism (v.9.1.1, GraphPad Software) with centre line depicting median unless otherwise stated. Analyses of epidemiological data and mutation and sequence data were performed in R (v.3.6.2. or v.4.1.3 (UKBB analysis)). Graphic display was performed in Prism, and illustrations in Fig. 3c,e and Extended Data Figs. 1a, 5d,f and 8a were created using BioRender (https://biorender.com). A Kolmogorov–Smirnov normality test was performed before any other statistical test. Afterwards, if any of the comparative groups failed normality (or the number was too low to estimate normality), a nonparametric Mann–Whitney test was performed. When groups showed a normal distribution, an unpaired two-tailed t-test was performed. When groups showed a significant difference in the variance, we used a t-test with Welch's correction. When assessing statistics of three or more groups, we performed ANOVA or nonparametric Kruskal–Wallis test controlling for multiple comparisons. Blinded analysis was carried out for all image and tumour analysis. No data were excluded. No statistical methods were used to predetermine sample sizes in the mouse studies. Mice with matched sex and age were randomized into different treatment groups. All experiments were reliably reproduced. Specifically, all in vivo experiments, except for omics data (RNA-seq), were performed independently at least twice, with the total number of biological replicates (independent mice) indicated in the corresponding figure legends.

## Driver mutation probability

The list of driver mutations and the mutational signature exposures were obtained from the TRACERx 421 publication^14^. Only patients with detected smoking-related signatures are considered in the analysis (TRACERx 421). Each observed clonal driver mutation was given a probability to be caused by all active mutational signatures in the patient. This number was derived by multiplying the exposures of the mutational signatures with the 96-channel profile of each signature^74^. Then the value was normalized to 1 so that each driver mutation can be explained by a fraction of active mutational signatures. The probabilities were then aggregated, giving the overall contribution to driver mutations from each of the active mutational signatures. A patient was defined as non-carrier of a tobacco-related driver mutation if the probability of SBS4 and SBS92 (smoking-related signatures) was less than 0.5.

## Extended Data

**Extended Data Figure 7:**
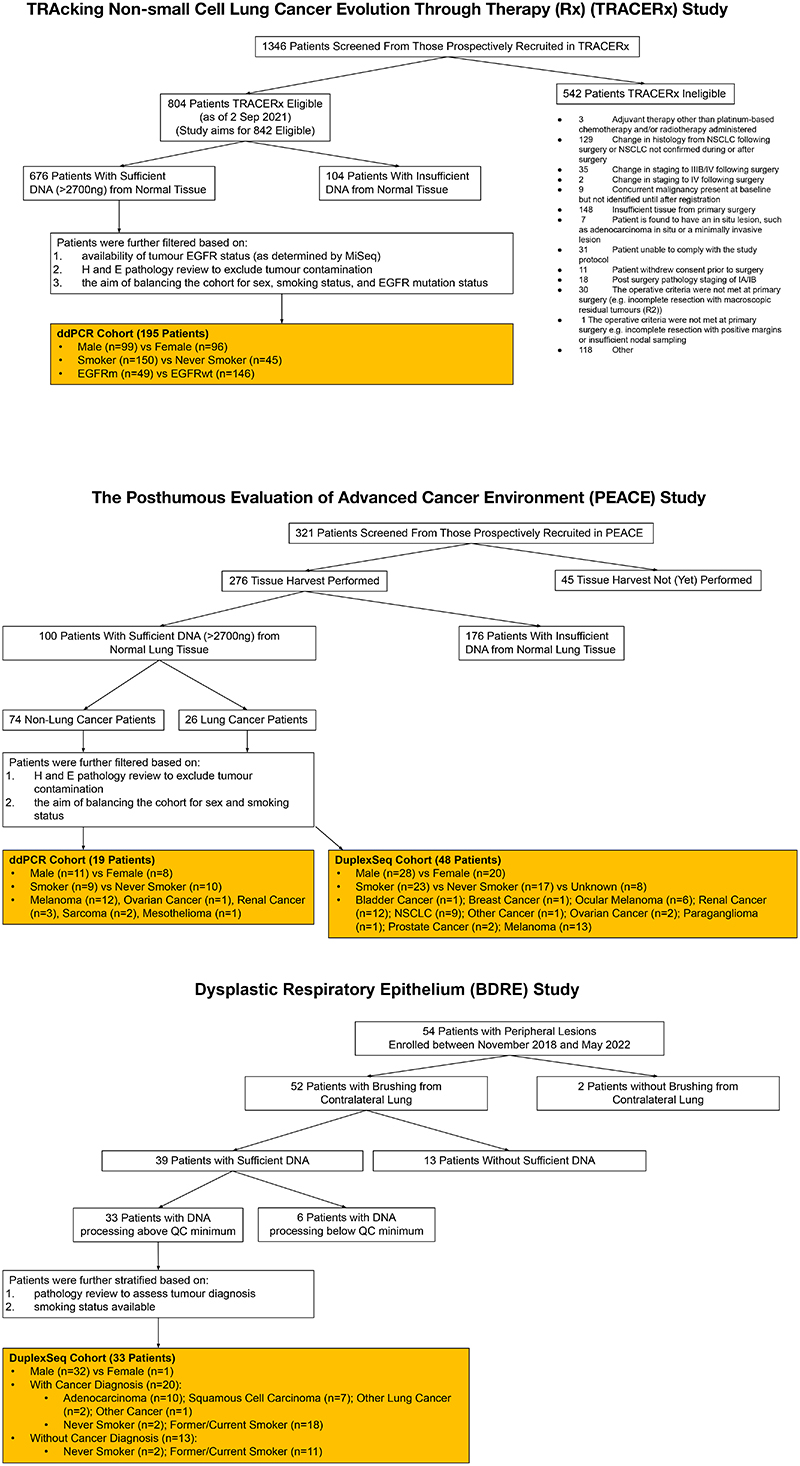
CONSORT Diagrams for the normal lung tissue profiling cohorts

**Extended Data Figure 8:**
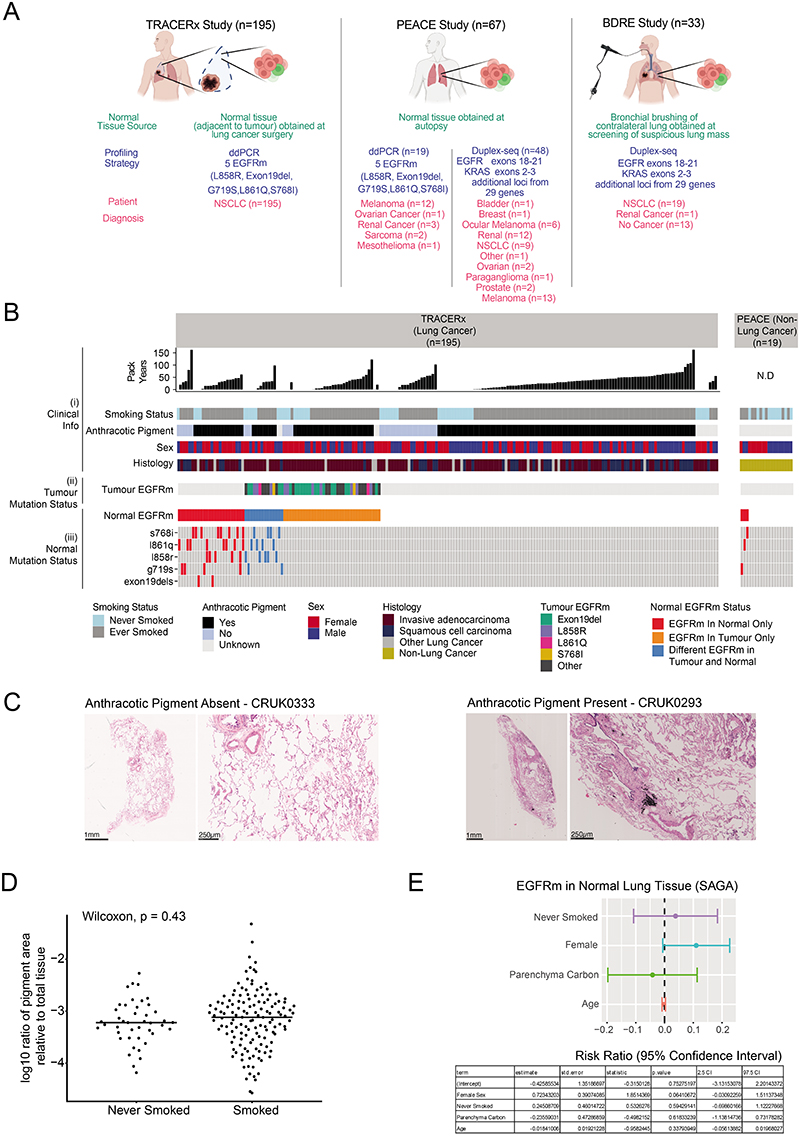
A) Schematic indicating normal lung tissue cohorts analysed by ddPCR and Duplex-seq. B) TRACERx and PEACE Cohort for ddPCR of 5 *EGFR* mutations. (i) Clinical information for each patient, (ii) Tumour *EGFR* mutation status, (iii) Normal *EGFR* mutation status. C) Representative H & E images from anthracotic pigment identification in TRACERx normal tissue. D) Comparing area of normal tissue harbouring anthracotic pigment in never smokers (n=43) and smokers (n=138). Each dot represents the ratio of pigmented area respective to total tissue in each anthracosis positive normal lung tissue sample. Two-sided Wilcox test p-value is reported. E) Regression analysis of characteristics influences *EGFR* mutant (EGFRm) presence in normal lung tissue for ddPCR-TRACERx cohort (n=195)

**Extended Data Figure 9:**
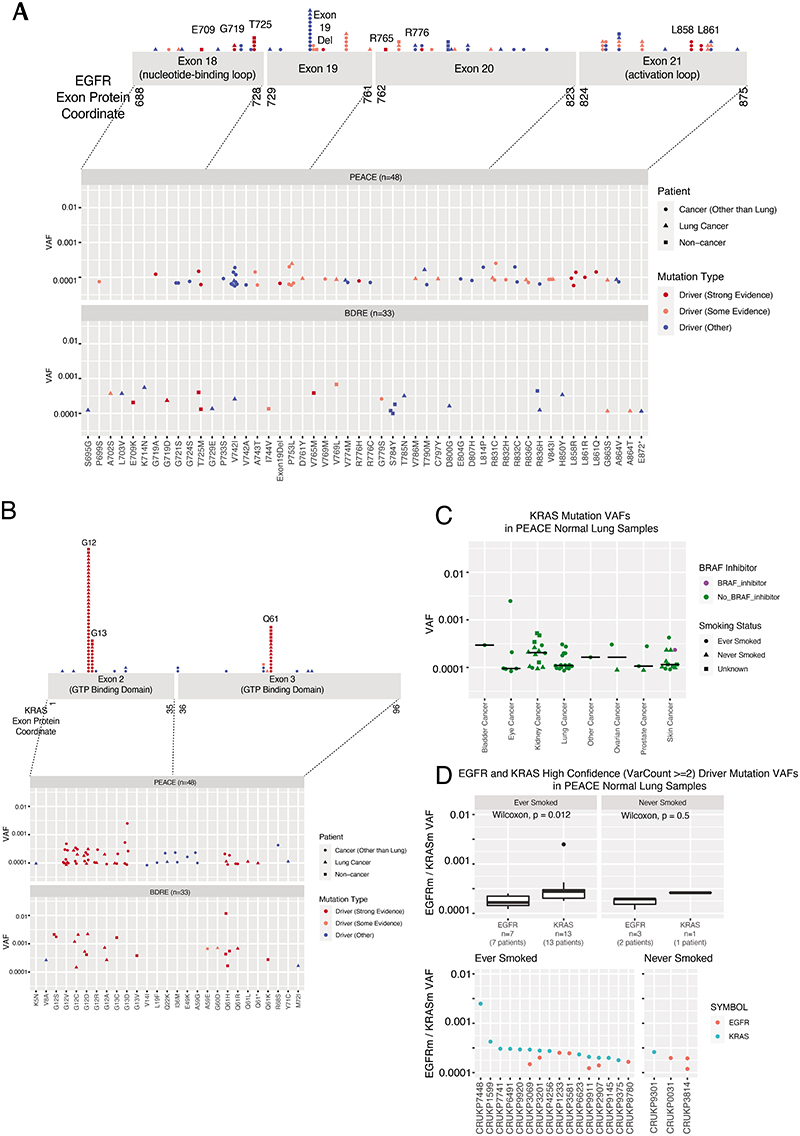
A) Top: *EGFR* Mutations detected using Duplex-seq across *EGFR* exons 18-21 on normal lung samples from the BDRE Study. Bottom: VAFs of each *EGFR* mutation are displayed. B) Top: *KRAS* Mutations detected using Duplex-seq across *KRAS* exons 2-3 on normal lung samples from the BDRE Study. Bottom: VAFs of each *KRAS* mutation are displayed. A-B) Only cancer-related mutations annotated in the cancer gene census are displayed. Mutations with strong evidence of being a lung cancer driver mutation are indicated in red, while mutations with some evidence of being a lung cancer driver mutation are indicated in pink, all other drivers annotated in COSMIC are indicated in blue. C) VAFs of *KRAS* mutations across samples of different cancer types. The one patient who received BRAF inhibitor treatment is indicated in purple. D) Comparing VAFs of high confidence (var count >=2, strong evidence) driver mutations in *EGFR* and *KRAS*. TOP: Boxplots summarise VAFs across samples. The boxplot line represents the median, the hinges of the box represent the 1st and 3rd quartiles and the limits of the whiskers represent the 1.5 interquartile range. Mutations are grouped according to the gene harbouring the mutation and smoking status of the patient. Two-sided Wilcox test p-values are reported. BOTTOM: dot plots show VAFs of mutations in each sample. Where a sample has 2 mutations (n=4), they are both indicated. Dots are coloured by the gene harbouring the mutation (*EGFR* or *KRAS*). A paired t-test was performed between the VAFs of *EGFR* and *KRAS* mutations in these 4 cases. (Paired t-test p=0.015) (Details of driver mutations can be found in Supplementary Table S8)

## Supplementary Material

Supplementary Table 1 England Cohort - Patient Characteristics Summary of clinical characteristics from the England lung cancer cohort. The “EGFRwt vs EGFRm” sheet compares patients with and without *EGFR* mutations in their tumours, while the “EGFRm Tested vs Non Tested” compares patients who were tested and untested for *EGFR* mutations. Chi-squared test p-values are reported.

Supplementary Table 2 South Korea Cohort - Patient Characteristics Summary of clinical characteristics from the South Korea lung cancer cohort. The “EGFRwt vs EGFRm” sheet compares patients with and without *EGFR* mutations in their tumours. Chi-squared test p-values are reported.

Supplementary Table 3 Taiwan Cohort - Patient Characteristics Summary of clinical characteristics from the Taiwan lung cancer cohort cohort. The “EGFRwt vs EGFRm” sheet compares patients with and without *EGFR* mutations in their tumours, while the “EGFRm Tested vs Non Tested” compares patients who were tested and untested for *EGFR* mutations. Chi-squared test p-values are reported.

Supplementary Table 4 UK Biobank Interaction Tests & Cancer Type Definitions Results of multivariable Cox regressions investigating PM2.5 and cancer incidence in the UK Biobank. The “Lung (main)” sheet shows the results for all covariates for the analysis on the full cohort. The “PanCancer” sheet features the results for PM2.5 for all cancer types analysed. The “Lung (adenocarcinoma only)” and “Lung (migration)” sheets contain the results for all covariates when analysing only lung adenocarcinoma and those who remained at their baseline address for at least three years prior to baseline, respectively. Cox-regression p-values are reported. The “ICD10codes_cancerTypes” sheet contains the International Classification of Diseases (ICD) 10 codes used to define each analysed cancer type.

Supplementary Table 5 Mouse RNA-seq Results of the differential expression analysis of RNA-seq libraries from reporter tdTomato mice exposed to PBS (T), or particulate matter (T+PM); or tdTomato;*EGFR*^L858R^ mice exposed to PBS (ET+PBS) or particulate matter (ET+PM). The “Mouse DGE Analysis” sheet features for each gene, metrics output from DESeq2 and the top 2 principal components from the PCA analysis. The “Mouse and Human DGE Analysis Comparison” sheet features for each gene where human and mouse orthologs can be mapped, the differential expression analysis metrics between air pollution exposed libraries (Mouse: T+PM; Human: Diesel Exhaust (DE)) vs control (Mouse: T; Human: Filtered Air (FA)) libraries.

Supplementary Table 6 Normal Tissue Datasets Analysed for *EGFR* Mutations Published datasets of DNA sequencing of normal human tissues (skin, lung, oesophagus, colorectal, small intestine, liver, uterus and bladder) describing patient cohorts, sampling, sequencing technology and mutation calling. The presence of any *EGFR* mutation and *EGFR*^L858R^ mutation with associated variant allele frequency (VAFs) are reported.

Supplementary Table 7 TRACERx, PEACE, BDRE Studies - Cohort Clinical Characteristics Clinical characteristics of each patient included in the normal lung tissue profiling work. There is one sheet dedicated to each cohort: “ddPCR - TRACERx”, “ddPCR - PEACE”, “Duplex-seq - PEACE” and “Duplex-seq BDRE”.

Supplementary Table 8 Evidence of *EGFR*/*KRAS* Cancer Driver Mutation Status Non-silent *EGFR* and *KRAS* mutations, detected by Duplex-Seq within PEACE and BDRE cohorts, were researched for published evidence of cancer driver status. For *EGFR*, literature reports of >1 patient with mutation (not including compound mutations) achieving Stable Disease (SD), Partial Response (PR) or Complete Response (CR) to a clinical *EGFR* inhibitor combined with supporting evidence (defined from *in vitro* studies, mouse model data or protein modelling analyses) were classified as ‘strong evidence’ drivers. Reports of 1 patient with SD, PR or CR to clinical *EGFR* inhibition OR the presence of other supporting evidence were defined as ‘some evidence’ drivers. No reported patient sensitivity to EGFR inhibition or little supporting reports were defined as ‘weak evidence’ drivers. For *KRAS*, literature reportsof frequent occurrence in cancer (above or equal to 2% of *KRAS* mutant cancers), *in vitro* studies and mouse model data were classified as ‘strong evidence’ drivers; protein modelling analyses were classified as ‘some evidence’ drivers.

Supplementary Table 9 Frequency of *EGFR* mutant and *KRAS* mutant In Normal Lung Tissue Summaries of the proportion of patients with normal lung tissues harbouring *EGFR* or KRAS mutations. Patients are stratified according to diagnosis, sex, and smoking status. Proportion test p-values are reported.

Supplementary Table 10 List of Antibodies Antibodies used for flow cytometry, immunohistochemical and immunofluorescence analyses of mouse tissue.

TRACERx Consortium

## Data Availability

Duplex-seq data for the PEACE and BDRE cohorts are available at the European Genome-Phenome Archive (EGA) with the identifier EGAS00001006951. Duplex-seq data generated from PEACE study samples during this study are not publicly available and restrictions apply to the availability of these data. Such Duplex-seq data are available through the Cancer Research UK and University College London Cancer Trials Centre (ctc.peace@ucl.ac.uk) for academic, non-commercial research purposes upon reasonable request and subject to review of a project proposal that will be evaluated by a PEACE data access committee, entering into an appropriate data access agreement and subject to any applicable ethical approvals. Duplex-seq data generated from the BDRE study are available through J. DeGregori (James.Degregori@cuanschutz.edu) for academic, non-commercial research purposes upon reasonable request, entering into an appropriate data access agreement and subject to any applicable ethical approvals. The Duplex-seq data for the BDRE and PEACE studies were generated using a larger panel of probes that covered approximately 50 kb of the genome, spanning hotspots frequently mutated in cancers. This full dataset has been provided for the 17 never-smoker individuals from the PEACE study. For all other samples, only data for the EGFR and KRAS regions queried are included in this manuscript. The RNA-seq data for the COPA study are available at the EGA with the identifier EGAS00001006966. De-identified participant data are available upon reasonable request to C.C. (christopher. carlsten@ubc.ca) for academic, non-commercial research purposes. Data availability is subject to a data access agreement and applicable ethical approvals. Mouse WGS data are available at the European Nucleotide Archive (ENA) with the identifier PRJEB58221 (ERP143287). Mouse RNA-seq data are available at the ENA with the identifier PRJEB59269 (ERP144330). Source data are provided with this paper.

## Code availability

Code for analysis of epidemiology, RNA-seq and WGS data and processing of healthy lung tissue are available at Zenodo (https://doi.org/10.5281/zenodo.7705022).
