# Supplementary material for: Lung adenocarcinoma promotion by air pollutants
Source: Nature. Author manuscript; Available in PMC 2023 Jun 1. (PMC7614604; doi:10.1038/s41586-023-05874-3)
Supplement: TRACERx Consortium [file EMS173669-supplement-TRACERx_Consortium.docx]

^1^Cancer Evolution and Genome Instability Laboratory, The Francis Crick Institute, London, UK. ^2^Cancer Research UK Lung Cancer Centre of Excellence, University College London Cancer Institute, London, UK. ^3^Division of Medicine, University College London, London, UK. ^4^Tumour Immunogenomics and Immunosurveillance Laboratory, University College London Cancer Institute, London, UK. ^5^Department of Thoracic Surgery and Thoracic Oncology Institute, Peking University People’s Hospital, Beijing, China. ^6^Department of Hematology and Oncology, Chang Gung Memorial Hospital, Chiayi Branch, Chiayi, Taiwan. ^7^Graduate Institute of Clinical Medical Sciences, Chang-Gung University, Taoyuan, Taiwan. ^8^Department of Biochemistry and Molecular Genetics, University of Colorado Anschutz Medical Campus, Aurora, CO, USA. ^9^Department of Biomedical Sciences, University of Cagliari, Cagliari, Italy. ^10^Department of Cellular Pathology, University College London Hospitals, London, UK. ^11^Tumour–Host Interaction Laboratory, The Francis Crick Institute, London, UK. ^12^Division of Hematology-Oncology, Department of Medicine, Samsung Medical Center, Sungkyunkwan University School of Medicine, Seoul, Korea. ^13^BC Cancer Research Institute, University of British Columbia, Vancouver, British Columbia, Canada. ^14^Oncogene Biology Laboratory, The Francis Crick Institute, London, UK. ^15^Department of Molecular Cell Biology and Immunology, Amsterdam UMC, Amsterdam, The Netherlands. ^16^Cancer Metastasis Laboratory, University College London Cancer Institute, London, UK. ^17^Cancer Genome Evolution Research Group, Cancer Research UK Lung Cancer Centre of Excellence, University College London Cancer Institute, London, UK. ^18^Department of Medicine, Division of Respiratory Medicine, Chan-Yeung Centre for Occupational and Environmental Respiratory Disease, Vancouver Coastal Health Research Institute, UBC, Vancouver, British Columbia, Canada. ^19^Ontario Institute for Cancer Research, Toronto, Ontario, Canada. ^20^Department of Pathobiology and Population Sciences, The Royal Veterinary College, Hatfield, UK. ^21^Experimental Histopathology, The Francis Crick Institute, London, UK. ^22^National Disease Registration Service (NDRS), NHS England, Leeds, UK. ^23^Centre for Cancer, Society and Public Health, Comprehensive Cancer Centre, School of Cancer and Pharmaceutical Sciences, King’s College London, London, UK. ^24^Health Information and Epidemiology Laboratory, Chang-Gung Memorial Hospital, Chiayi, Taiwan. ^25^Flagship Biosciences, Boulder, CO, USA. ^26^Division of Pulmonary Sciences and Critical Care Medicine, Department of Medicine,

University of Colorado Anschutz Medical Campus, Aurora, CO, USA. ^27^Veterans Affairs Eastern Colorado Healthcare System, Aurora, CO, USA. ^28^Department of Pathology, University of Colorado Anschutz Medical Campus, Aurora, CO, USA. ^29^SAGA Diagnostics, Lund, Sweden. ^30^Division of Oncology, Department of Clinical Sciences, Lund University, Lund, Sweden. ^31^Early Cancer Detection Consultant, Bethesda, MD, USA. ^32^Comprehensive Cancer Centre, King’s College London, London, UK. ^33^Division of Genetics and Epidemiology, Institute of Cancer Research, London, UK. ^34^David H. Koch Institute for Integrative Cancer Research, Cambridge, MA, USA. ^35^Department of Biology, Massachusetts Institute of Technology, Cambridge, MA, USA. ^36^Cancer Research UK and UCL Cancer Trials Centre, London, UK. ^37^Department of Oncology, University College London Hospitals, London, UK. ^126^These authors contributed equally: William Hill, Emilia L. Lim, Clare E. Weeden. ^127^These authors jointly supervised this work: Emilia L. Lim, James DeGregori, Mariam Jamal-Hanjani. *A list of authors and their affiliations appears at the end of the paper. ^128^Corresponding author: [Charles.Swanton@crick.ac.uk](mailto:Charles.Swanton@crick.ac.uk)

TRACERx Consortium

Jason F. Lester^38^, Amrita Bajaj^39^, Apostolos Nakas^39^, Azmina Sodha-Ramdeen^39^, Keng Ang^39^, Mohamad Tufail^39^, Mohammed Fiyaz Chowdhry^39^, Molly Scotland^39^, Rebecca Boyles^39^,Sridhar Rathinam^39^, Claire Wilson^40^, Domenic Marrone^40^, Sean Dulloo^40^, Dean A. Fennell^39,40^, Gurdeep Matharu^41^, Jacqui A. Shaw^41^, Joan Riley^41^, Lindsay Primrose^41^, Ekaterini Boleti^42^, Heather Cheyne^43^, Mohammed Khalil^43^, Shirley Richardson^43^, Tracey Cruickshank^43^, Gillian Price^44,45^, Keith M. Kerr^45,46^, Sarah Benafif^38^, Kayleigh Gilbert^47^, Babu Naidu^48^, Akshay J. Patel^49^, Aya Osman^49^, Christer Lacson^49^, Gerald Langman^49^, Helen Shackleford^49^, Madava Djearaman^49^, Salma Kadiri^49^, Gary Middleton^49,50^, Angela Leek^51^, Jack Davies Hodgkinson^51^, Nicola Totten^51^, Angeles Montero^52^, Elaine Smith^52^, Eustace Fontaine^52^, Felice Granato^52^, Helen Doran^52^, Juliette Novasio^52^, Kendadai Rammohan^52^, Leena Joseph^52^, Paul Bishop^52^, Rajesh Shah^52^, Stuart Moss^52^, Vijay Joshi^52^, Philip Crosbie^52,53,54^, Fabio Gomes^55^, Kate Brown^55^, Mathew Carter^55^, Anshuman Chaturvedi^54,55^, Lynsey Priest^54,55^, Pedro Oliveira^54,55^, Colin R. Lindsay^56^, Fiona H. Blackhall^56^, Matthew G. Krebs^56^, Yvonne Summers^56^, Alexandra Clipson^54,57^, Jonathan Tugwood^54,57^, Alastair Kerr^54,57^, Dominic G. Rothwell^54,57^, ElaineKilgour^54,57^,Caroline Dive^54,57^, Hugo J. W. L. Aerts^58,59,60^, Roland F. Schwarz^61,62^, Tom L. Kaufmann^62,63^, Gareth A. Wilson^1^, Rachel Rosenthal^1^, Peter Van Loo^64,65,66^, Nicolai J. Birkbak^1,2,67,68,69^,Zoltan Szallasi^70,71,72^, Judit Kisistok^67,68,69^, Mateo Sokac^67,68,69^, Roberto Salgado^73,74^, Miklos Diossy^70,71,75^, Jonas Demeulemeester^66,76,77^, Abigail Bunkum^2,16,78^, Aengus Stewart^79^,Alastair Magness^1,2^, Alexander M. Frankell^1,2^, Andrew Rowan^1^, Angeliki Karamani^80^, Antonia Toncheva^2^, Ariana Huebner^1,2,17^, Benny Chain^80^, Brittany B. Campbell^1^, CarlaCastignani^66,81^, Carlos Martínez-Ruiz^2,17^, Charles Swanton^1,2,37^, Chris Bailey^1^, Christopher Abbosh^2^, Clare Puttick^1,2,17^, Clare E. Weeden^1,126^, Claudia Lee^1,2,3^, Corentin Richard^2^, Crispin T.Hiley^1,2^, Cristina Naceur-Lombardelli^2^, David A. Moore^1,2,10^, David R. Pearce^80^, Despoina Karagianni^80^, Dhruva Biswas^1,2,82^, Dina Levi^79^, Elena Hoxha^80^, Elizabeth Larose Cadieux^66,81^,Emilia L. Lim^1,2,126,127^, Emma Colliver^1^, Emma Nye^21^, Eva Grönroos^1^, Felip Gálvez-Cancino^80^, Foteini Athanasopoulou^1,2,83^, Francisco Gimeno-Valiente^2^, George Kassiotis^84,85^, GeorgiaStavrou^80^,Gerasimos Mastrokalos^80^, Haoran Zhai^1,2^, Helen L. Lowe^80^, Ignacio Garcia Matos^80^, Jacki Goldman^79^, James L. Reading^80^, James R. M. Black^2,17^, Javier Herrero^82^,Jayant K. Rane^1,80^, Jerome Nicod^83^, Jie Min Lam^2,16,38^, John A. Hartley^80^, Karl S. Peggs^86,87^, Katey S. S. Enfield^1^, Kayalvizhi Selvaraju^80^, Kerstin Thol^2,17^, Kevin Litchfield^2,4^, Kevin W.Ng^84^,Kezhong Chen^2,5^, Krijn Dijkstra^88,89^, Kristiana Grigoriadis^1,2,17^, Krupa Thakkar^2^, Leah Ensell^80^, Maise Al Bakir^1,2^, Mansi Shah^80^, Marcos Vasquez Duran^80^, Maria Litovchenko^80^,Mariam Jamal-Hanjani^2,16,37,127^, Mariana Werner Sunderland^2^, Mark S. Hill^1^, Michelle Dietzen^1,2,17^, Michelle Leung^1,2,17^, Mickael Escudero^79^, Mihaela Angelova^1^,Miljana Tanić^81,90^, Monica Sivakumar^2^, Nicholas McGranahan^2,17^, Nnennaya Kanu^2^, Olga Chervova^80^, Olivia Lucas^1,2,38,78^, Oriol Pich^1^, Othman Al-Sawaf^1,2,91^, Paulina Prymas^2^,Philip Hobson^79^, Piotr Pawlik^80^, Richard Kevin Stone^21^, Robert Bentham^2,17^, Robert E. Hynds^80^, Roberto Vendramin^79^, Sadegh Saghafinia^2^, Saioa López^80^,Samuel Gamble^80^, Selvaraju Veeriah^2^, Seng Kuong Anakin Ung^80^, Sergio A. Quezada^2,92^, Sharon Vanloo^2^, Simone Zaccaria^2,78^, Sonya Hessey^2,16,78^, Sophia Ward^1,2,83^, Stefan Boeing^79^,Stephan Beck^81^, Supreet Kaur Bola^80^, Takahiro Karasaki^1,2,16^, Tamara Denner^79^, Teresa Marafioti^10^, Thanos P. Mourikis^80^, Thomas B. K. Watkins^1^, Victoria Spanswick^80^,Vittorio Barbè^79^, Wei-Ting Lu^79^, William Hill^1,126^, Wing Kin Liu^2,16^, Yin Wu^80^, Yutaka Naito^79^,Zoe Ramsden^79^, Catarina Veiga^93^, Gary Royle^94^, Charles-Antoine Collins-Fekete^95^, Francesco Fraioli^96^, Paul Ashford^97^, Tristan Clark^98^, Martin D.Forster^2,38^, Siow Ming Lee^2,99^,Elaine Borg^10^, Mary Falzon^10^, Dionysis Papadatos-Pastos^38^, James Wilson^38^, Tanya Ahmad^38^, Alexander James Procter^100^, Asia Ahmed^100^, Magali N. Taylor^100^, Arjun Nair^100,101^,David Lawrence^102^, Davide Patrini^102^, Neal Navani^103,104^, Ricky M. Thakrar^103,104^, Sam M. Janes^103^, Emilie Martinoni Hoogenboom^105^, Fleur Monk^105^, James W. Holding^105^,Junaid Choudhary^105^, Kunal Bhakhri^105^, Marco Scarci^105^, Martin Hayward^105^, Nikolaos Panagiotopoulos^105^, Pat Gorman^105^, Reena Khiroya^10^, Robert C. M. Stephens^105^,Yien Ning Sophia Wong^105^, Steve Bandula^105^, Allan Hackshaw^36^, Abigail Sharp^36^, Sean Smith^36^, Nicole Gower^36^, Harjot Kaur Dhanda^36^, Kitty Chan^36^, Camilla Pilotti^36^,Rachel Leslie^36^, Anca Grapa^106^, Hanyun Zhang^106^, Khalid AbdulJabbar^106^, Xiaoxi Pan^106^, Yinyin Yuan^107^, David Chuter^108^, Mairead MacKenzie^108^, Serena Chee^109^, Aiman Alzetani^109^,Judith Cave^110^, Lydia Scarlett^109^, Jennifer Richards^109^, Papawadee Ingram^109^, Silvia Austin^109^, Eric Lim^111,112^, Paulo De Sousa^112^, Simon Jordan^112^, Alexandra Rice^112^, HilgardtRaubenheimer^112^, Harshil Bhayani^112^, Lyn Ambrose^112^, Anand Devaraj^112^, Hema Chavan^112^, Sofina Begum^112^, Silviu I. Buderi^112^, Daniel Kaniu^112^, Mpho Malima^112^, Sarah Booth^112^, AndrewG. Nicholson^113,114^, Nadia Fernandes^112^, Pratibha Shah^112^, Chiara Proli^112^, Madeleine Hewish^115,116^, Sarah Danson^117^, Michael J. Shackcloth^118^, Lily Robinson^119^, Peter Russell^119^, Kevin G. Blyth^120,121,122^, Craig Dick^123^, John Le Quesne^120,121,124^, Alan Kirk^125^, Mo Asif^125^, Rocco Bilancia^125^, Nikos Kostoulas^125^ & Mathew Thomas^125^

^38^Singleton Hospital, Swansea Bay University Health Board, Swansea, UK. ^39^University Hospitals of Leicester NHS Trust, Leicester, UK.^40^University of Leicester, Leicester, UK.^41^Cancer Research Centre, University of Leicester, Leicester, UK. ^42^Royal Free Hospital, Royal Free London NHS Foundation Trust, London, UK. ^43^Aberdeen Royal Infirmary NHS Grampian, Aberdeen, UK. ^44^Department of Medical Oncology, Aberdeen Royal Infirmary NHS Grampian, Aberdeen, UK. ^45^University of Aberdeen, Aberdeen, UK. ^46^Department of Pathology, Aberdeen Royal Infirmary NHS Grampian, Aberdeen, UK. ^47^The Whittington Hospital NHS Trust, London, UK. ^48^Birmingham Acute Care Research Group, Institute of Inflammation and Ageing, University of Birmingham, Birmingham, UK. ^49^University Hospital Birmingham NHS Foundation Trust, Birmingham, UK. ^50^Institute of Immunology and Immunotherapy, University of Birmingham, Birmingham, UK. ^51^Manchester Cancer Research Centre Biobank, Manchester, UK. ^52^Wythenshawe Hospital, Manchester University NHS Foundation Trust, Wythenshawe, UK. ^53^Division of Infection, Immunity and Respiratory Medicine, University of Manchester, Manchester, UK. ^54^Cancer Research UK Lung Cancer Centre of Excellence, University of Manchester, Manchester, UK. ^55^The Christie NHS Foundation Trust, Manchester, UK. ^56^Division of Cancer Sciences, The University of Manchester and The Christie NHS Foundation Trust, Manchester, UK. ^57^Cancer Research UK Manchester Institute Cancer Biomarker Centre, University of Manchester, Manchester, UK. ^58^Artificial Intelligence in Medicine (AIM) Program, Mass General Brigham, Harvard Medical School, Boston, MA, USA. ^59^Department of Radiation Oncology, Brigham and Women’s Hospital, Dana-Farber Cancer Institute, Harvard Medical School, Boston, MA, USA. ^60^Radiology and Nuclear Medicine, CARIM and GROW, Maastricht University, Maastricht, The Netherlands. ^61^Institute for Computational Cancer Biology, Center for Integrated Oncology (CIO), Cancer Research Center Cologne Essen (CCCE), Faculty of Medicine and University Hospital Cologne, University of Cologne, Cologne, Germany. ^62^Berlin Institute for the Foundations of Learning and Data (BIFOLD), Berlin, Germany. ^63^Berlin Institute for Medical Systems Biology, Max DelbrückCenter for Molecular Medicine in the Helmholtz Association (MDC), Berlin, Germany. ^64^Department of Genetics, The University of Texas MDAnderson Cancer Center, Houston, TX, USA. ^65^Department of Genomic Medicine, The University of Texas MD Anderson Cancer Center, Houston, TX, USA. ^66^Cancer Genomics Laboratory, The Francis Crick Institute, London, UK. ^67^Department of Molecular Medicine, Aarhus University Hospital, Aarhus, Denmark. ^68^Department of Clinical Medicine, Aarhus University, Aarhus, Denmark. ^69^Bioinformatics Research Centre, Aarhus University, Aarhus, Denmark. ^70^Danish Cancer Society Research Center, Copenhagen, Denmark.^71^ComputationalHealth Informatics Program, Boston Children’s Hospital, Boston, MA, USA. ^72^Department of Bioinformatics, Semmelweis University, Budapest, Hungary. ^73^Department of Pathology, ZAS Hospitals, Antwerp, Belgium. ^74^Division of Research, Peter MacCallum Cancer Centre, Melbourne, Victoria, Australia. ^75^Department of Physics of Complex Systems, ELTE E.tv.sLornd University, Budapest, Hungary. ^76^Integrative Cancer Genomics Laboratory, Department of Oncology, KU Leuven, Leuven, Belgium. ^77^VIB–KU Leuven Center for CancerBiology, Leuven, Belgium. ^78^Computational Cancer Genomics Research Group, University College London Cancer Institute, London, UK. ^79^The Francis Crick Institute, London, UK.^80^University College London Cancer Institute, London, UK. ^81^Medical Genomics, University College London Cancer Institute, London, UK. ^82^Bill Lyons Informatics Centre, UniversityCollege London Cancer Institute, London, UK. ^83^Advanced Sequencing Facility, The Francis Crick Institute, London, UK. ^84^Retroviral Immunology Group, The Francis Crick Institute, London, UK. ^85^Department of Infectious Disease, Faculty of Medicine, Imperial College London, London, UK. ^86^Department of Haematology, University College London Hospitals,London, UK. ^87^Cancer Immunology Unit, Research Department of Haematology, University College London Cancer Institute, London, UK. ^88^Department of Molecular Oncology andImmunology, the Netherlands Cancer Institute, Amsterdam, The Netherlands. ^89^Oncode Institute, Utrecht, The Netherlands. ^90^Experimental Oncology, Institute for Oncology andRadiology of Serbia, Belgrade, Serbia. ^91^Cancer Metastasis Laboratory, University College London Cancer Institute, London, UK. ^92^Immune Regulation and Tumour ImmunotherapyGroup, Cancer Immunology Unit, Research Department of Haematology, University College London Cancer Institute, London, UK. ^93^Centre for Medical Image Computing, Department of Medical Physics and Biomedical Engineering, University College London, London, UK. ^94^Department of Medical Physics and Bioengineering, University College London CancerInstitute, London, UK. ^95^Department of Medical Physics and Biomedical Engineering, University College London, London, UK. ^96^Institute of Nuclear Medicine, Division of Medicine,University College London, London, UK. ^97^Institute of Structural and Molecular Biology, University College London, London, UK. ^98^University College London, London, UK. ^99^Department of Oncology, University College London Hospitals, London, UK. ^100^Department of Radiology, University College London Hospitals, London, UK. ^101^UCL Respiratory,Department of Medicine, University College London, London, UK. ^102^Department of Thoracic Surgery, University College London Hospital NHS Trust, London, UK. ^103^Lungs for LivingResearch Centre, UCL Respiratory, University College London, London, UK. ^104^Department of Thoracic Medicine, University College London Hospitals, London, UK. ^105^University CollegeLondon Hospitals, London, UK. ^106^The Institute of Cancer Research, London, UK. ^107^TheUniversity of Texas MD Anderson Cancer Center, Houston, TX, USA. ^108^Independent Cancer Patients’ Voice, London, UK. ^109^University Hospital Southampton NHS Foundation Trust, Southampton, UK. ^110^Department of Oncology, University Hospital Southampton NHS Foundation Trust, Southampton, UK. ^111^Academic Division of Thoracic Surgery, Imperial College London, London, UK. ^112^Royal Brompton and Harefield Hospitals, Guy’s and StThomas’ NHS Foundation Trust, London, UK. ^113^Department of Histopathology, Royal Brompton and Harefield Hospitals, Guy’s and St Thomas’ NHS Foundation Trust, London, UK. ^114^National Heart and Lung Institute, Imperial College London, London, UK. ^115^Royal Surrey Hospital, Royal Surrey Hospitals NHS Foundation Trust, Guilford, UK. ^116^University of Surrey, Guilford, UK. ^117^Sheffield Teaching Hospitals NHS Foundation Trust, Sheffield, UK. ^118^LiverpoolHeart and Chest Hospital, Liverpool, UK. ^119^Princess Alexandra Hospital, The Princess Alexandra Hospital NHS Trust, Harlow, UK. ^120^School of Cancer Sciences, University of Glasgow, Glasgow, UK. ^121^Cancer Research UK Beatson Institute, Glasgow, UK. ^122^QueenElizabeth University Hospital, Glasgow, UK. ^123^NHS Greater Glasgow and Clyde, Glasgow, UK.^124^Pathology Department, Queen Elizabeth University Hospital, NHS Greater Glasgow and Clyde, Glasgow, UK. ^125^Golden Jubilee National Hospital, Clydebank, UK.
